# H2AX promotes replication fork degradation and chemosensitivity in BRCA-deficient tumours

**DOI:** 10.1038/s41467-024-48715-1

**Published:** 2024-05-24

**Authors:** Diego Dibitetto, Martin Liptay, Francesca Vivalda, Hülya Dogan, Ewa Gogola, Martín González Fernández, Alexandra Duarte, Jonas A. Schmid, Morgane Decollogny, Paola Francica, Sara Przetocka, Stephen T. Durant, Josep V. Forment, Ismar Klebic, Myriam Siffert, Roebi de Bruijn, Arne N. Kousholt, Nicole A. Marti, Martina Dettwiler, Claus S. Sørensen, Jean-Christophe Tille, Manuela Undurraga, Intidhar Labidi-Galy, Massimo Lopes, Alessandro A. Sartori, Jos Jonkers, Sven Rottenberg

**Affiliations:** 1 Institute of Animal Pathology, Vetsuisse Faculty, University of Bern, 3012 Bern, Switzerland; 2 Cancer Therapy Resistance Cluster and Bern Center for Precision Medicine, Department for Biomedical Research, University of Bern, 3012 Bern, Switzerland; 3 Institute of Molecular Cancer Research, University of Zürich, Zürich, Switzerland; 4 Division of Molecular Pathology, The Netherlands Cancer Institute, 1066CX Amsterdam, The Netherlands; 5 Oncode Institute, Amsterdam, The Netherlands; 6 DDR Biology, Bioscience, Oncology R&D, AstraZeneca, Cambridge, CB4 0WG UK; 7 Biotech Research and Innovation Centre, University of Copenhagen, 2200 N Copenhagen, Denmark; 8 Division of Clinical Pathology, Department of Diagnostics, Hôpitaux Universitaires de Genève, Geneva, Switzerland; 9 Division of Gynecology, Department of Pediatrics and Gynecology, Hôpitaux Universitaires de Genève, Geneva, Switzerland; 10 Faculty of Medicine, Department of Medicine and Center of Translational Research in Onco-Hematology, University of Geneva, Swiss Cancer Center Leman, Geneva, Switzerland; 11 Department of Oncology, Hôpitaux Universitaires de Genève, 4, Rue Gabrielle Perret-Gentil, Geneva, 1205 Switzerland; 12 Present Address: Department of Experimental Oncology, Istituto di Ricerche Farmacologiche Mario Negri IRCCS, via Mario Negri 2, 20156 Milan, Italy

**Keywords:** Cancer therapeutic resistance, Stalled forks

## Abstract

Histone H2AX plays a key role in DNA damage signalling in the surrounding regions of DNA double-strand breaks (DSBs). In response to DNA damage, H2AX becomes phosphorylated on serine residue 139 (known as γH2AX), resulting in the recruitment of the DNA repair effectors 53BP1 and BRCA1. Here, by studying resistance to poly(ADP-ribose) polymerase (PARP) inhibitors in BRCA1/2-deficient mammary tumours, we identify a function for γH2AX in orchestrating drug-induced replication fork degradation. Mechanistically, γH2AX-driven replication fork degradation is elicited by suppressing CtIP-mediated fork protection. As a result, H2AX loss restores replication fork stability and increases chemoresistance in BRCA1/2-deficient tumour cells without restoring homology-directed DNA repair, as highlighted by the lack of DNA damage-induced RAD51 foci. Furthermore, in the attempt to discover acquired genetic vulnerabilities, we find that ATM but not ATR inhibition overcomes PARP inhibitor (PARPi) resistance in H2AX-deficient tumours by interfering with CtIP-mediated fork protection. In summary, our results demonstrate a role for H2AX in replication fork biology in BRCA-deficient tumours and establish a function of H2AX separable from its classical role in DNA damage signalling and DSB repair.

## Abstract (web summary)

Histone H2AX has a known role in DNA damage repair but interestingly, its loss is associated with resistance to poly(ADP-ribose) polymerase (PARP) inhibition in BRCA-deficient tumours. Here, the authors identify a role of γH2AX in the degradation of replication forks and demonstrate that H2AX loss drives PARP inhibitor resistance via increased stressed fork stability in BRCA-deficient tumours.

## Introduction

The advent of poly(ADP-ribose) polymerase inhibitors (PARPi) has revolutionized the treatment landscape for cancers harboring homologous recombination (HR) deficiencies, particularly those resulting from *BRCA1* and *BRCA2* mutations^1^. These mutations impair the cell’s ability to repair DNA double-strand breaks (DSBs) via HR^2^, thus compromising genomic stability. PARPi exploit this vulnerability by inducing synthetic lethality in BRCA-deficient cells^3,4^, which are unable to repair DNA damage effectively. Despite the clinical successes of PARPi, resistance invariably arises, posing significant challenges to durable therapeutic outcomes. The mechanisms underlying this resistance, which range from mutations restoring BRCA activity or HR to alterations in drug efflux mechanisms^5^, continue to be a focal point of research. Recent studies have challenged the prevailing model of PARPi action which posits that lethality arises primarily through “trapping” PARP enzymes onto DNA^6–8^. Indeed, work by Petropoulos et al. ^9^. suggests that the disruption of transcription-replication conflicts may play a pivotal role, indicating that our understanding of PARPi mechanisms is still evolving.

Functional CRISPR-Cas9 screens have emerged as powerful tools to identify mechanisms of drug resistance, including resistance to PARP inhibitors. By enabling the systematic knockout of genes across the genome in a high-throughput manner, these screens allow to uncover genes that, when lost, confer survival advantages to cancer cells under treatment pressure. This approach has proven instrumental in delineating the complex networks of genetic interactions and pathways that contribute to the emergence of resistance, providing valuable insights that can guide the development of next-generation therapies and combination treatments to circumvent resistance. The study of resistance mechanisms requires robust in vivo models that can recapitulate tumour dynamics and drug responses accurately. In this context, genetically engineered mouse models (GEMMs) of BRCA1- and BRCA2-associated breast cancer have proven invaluable advantages^5,10,11^. These models, including those developed with large intragenic deletions in *Brca1* or *Brca2* that preclude reactivation of protein function after secondary mutations, allow for the detailed investigation of PARPi resistance mechanisms that are BRCA-independent. For instance, these models have elucidated distinct patterns of resistance arising in BRCA1- versus BRCA2-deficient tumours^12^, such as the loss of 53BP1-RIF1-REV7-CST-SHLD complex driving HR restoration in BRCA1-deficient tumours^13–17^, and the loss of PARG leading to restoration of PARP signaling in BRCA2-deficient tumour^18^. Moreover, we and others have previously identified the restoration of replication fork protection as a key mechanism to protect genome stability and acquire chemotherapy resistance in BRCA-deficient tumour cells^19,20^. However, the precise genetic alterations in chemoresistant tumours underpinning restored fork protection have not been identified.

In this work, we carry out multiple functional CRISPR-Cas9 genetic screens and identify a critical role for the histone H2AX in mediating chemotherapy response. Importantly, we identify a role of H2AX in modulating replication fork stability which appears to contribute to the sensitivity of BRCA-deficient tumours to PARPi. By examining these dynamics, we find that γH2AX-dependent replication fork degradation is triggered by the inhibition of CtIP-mediated fork protection. Hence, we uncover additional molecular insights into both the mechanism of action of PARPi and the molecular basis of therapy resistance. This work promises to not only enhance our understanding of DNA damage response in cancer therapy but also to guide the development of more effective strategies to overcome resistance in *BRCA*-mutated cancers.

## Results

### Functional genetic screens and complementary in vivo analysis using BRCA1/2 models identify H2AX as a critical mediator of chemotherapy response

**Fig. 1:**
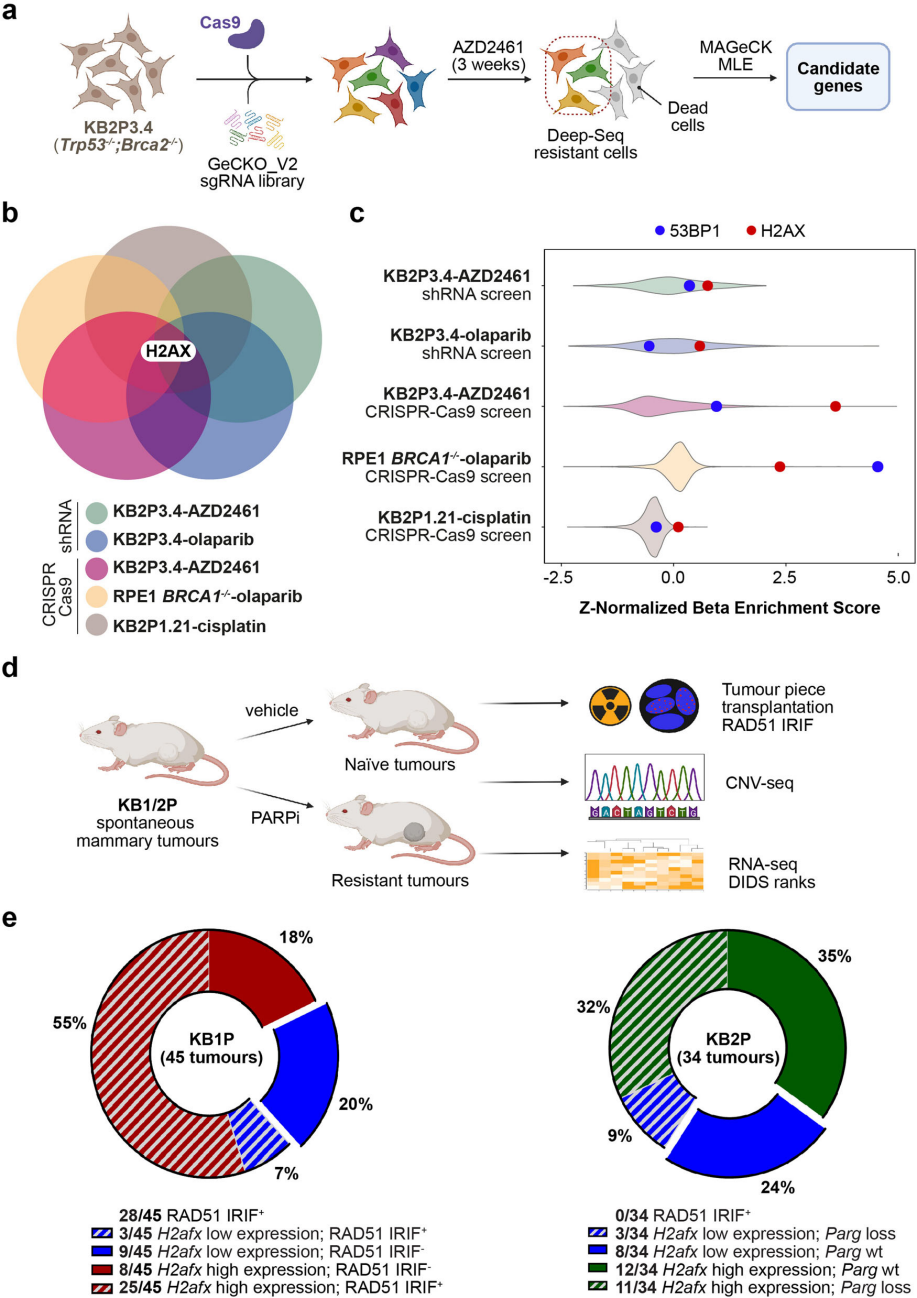
H2AX loss is frequently observed in BRCA-deficient mammary tumours with acquired PARPi resistance. **a** Design of the genome-wide CRISPR-Cas9 genetic screen carried out in KB2P3.4 cells treated with the poly(ADP-ribose) polymerase (PARP) inhibitor AZD2461. **b** Venn diagram showing the overlap of potential gene candidates identified in each individual screen. **c** Violin plots showing the Z-Normalized Beta-Enrichment Score from 5 different genetic screens for chemoresistance carried out in different BRCA1/2-deficient cell lines. Data were all analyzed using the MAGeCK MLE algorithm to allow for cross comparison. **d** Design of an in vivo pipeline to query for genetic alterations and Homologous Recombination (HR) restoration in PARPi-resistant mammary tumours from KB1/2P mice. **e** Pie charts showing 45 and 34 PARPi-resistant mammary tumours from KB1P and KB2P mice, respectively. Tumours are grouped based on RAD51 IRIF+, Parg mutational status and H2afx gene expression (see Methods text).

To identify new genes involved in the cellular response to PARPi in BRCA2;p53-deficient tumours, we performed a genome-wide CRISPR-Cas9 screen in our KB2P3.4 (*K14cre*;*Trp53*^*-/-*^*;Brca2*^*-/-*^) mouse mammary tumour cell line^21^. Cells were transduced with the mouse sgRNA GeCKO_V2 library targeting 20,628 genes^22^, and then treated with a nearly lethal dose of 200 nM of the AZD2461 PARPi for 3 weeks (Fig. 1a). At the end of the treatment, extracted genomic DNA from surviving cells was subjected to NGS and analyzed with the MAGeCK MLE algorithm^23^ (Fig. 1a). To increase the confidence of our screening data, we crossed the hits of this screen with the results obtained from four other genetic screens: two screens for PARPi resistance carried with a targeted DDR shRNA library in KB2P3.4 cells treated with AZD2461 or olaparib^18^; a genome-wide CRISPR-Cas9 screen performed in human RPE1-h*TERT TP53*^*-/-*^*;BRCA1*^*-/-*^ cells selected with olaparib^16^; a genome-wide CRISPR-Cas9 screen performed in KB2P1.21 cells (*K14cre*;*Trp53*^*-/-*^*;Brca2*^*-/-*^) treated with a lethal dose of cisplatin (Fig. 1b). This data processing allowed us to identify general chemoresistance mechanisms independent of: (1) the specific PARPi, (2) BRCA1 or BRCA2 deficiency, (3) the type of screen (shRNA- or CRISPR-based), (4) the species (mouse or human), or (5) the anti-cancer agent used (PARPi or cisplatin). Our analysis revealed that sgRNA/shRNA against the histone *H2afx*/*H2AFX* were greatly enriched in all the analyzed screens and scored among the top identified hits (Fig. 1b, c). Conversely, sgRNA against 53BP1, a known key modulator of chemoresistance in BRCA1-deficient tumours, were enriched specifically in the RPE1*-*h*TERT TP53*^*-/-*^*;BRCA1*^*-/-*^ screen but not in the screens performed in BRCA2-deficient cells (Fig. 1c). These results suggest that H2AX loss is associated with PARPi resistance through a mechanism different than 53BP1 inactivation which only occurs in BRCA1- but not in BRCA2-deficient cells^24^.

Considering the key role for γH2AX in DNA damage signalling^25,26^, it was surprising that *H2afx* loss would promote PARPi resistance. For this reason, we tested whether reduced *H2afx* gene expression would also occur in two cohorts of PARPi-resistant mammary tumours from KB1P and KB2P mice (*K14cre*;*Trp53*^*F/F*^*;Brca1*^*F/F*^ *and K14cre*;*Trp53*^*F/F*^*;Brca2*^*F/F*^, respectively), that acquired resistance in vivo following repeated PARPi cycles^12,18^ (Fig. 1d). In these tumours, we also determined RAD51 ionizing radiation-induced foci (IRIF) to distinguish Homologous Recombination (HR)-dependent from -independent mechanisms of PARPi resistance. We previously reported that none of the PARPi-resistant BRCA2-deficient tumours restored RAD51 IRIF^12^, whereas 64% (29/45) of the BRCA1-deficient tumours became RAD51 IRIF^+^ (Fig. 1e). Conversely, in the KB2P cohort, a large fraction of the tumours analyzed presented a major genomic structural change in the *Parg* locus, leading to the loss of *Parg* gene product (14/34) (Fig. 1e). Strikingly, we noticed that in both BRCA1/2-deficient tumour cohorts, *H2afx* gene expression was significantly reduced in a large fraction of the tumours (11/34 in the KB2P tumours and 12/45 in the KB1P tumours) with only a small overlap with *Parg* loss or HR restoration (Fig. 1e), which can be explained by the intra-tumoural heterogeneity of the resistance mechanisms^11,27^. Therefore, we concluded that *H2afx* downregulation frequently occurs in PARPi-resistant mammary tumours and that the underlying mechanism is likely to be different from previously reported mechanisms of resistance.

**Fig. 2:**
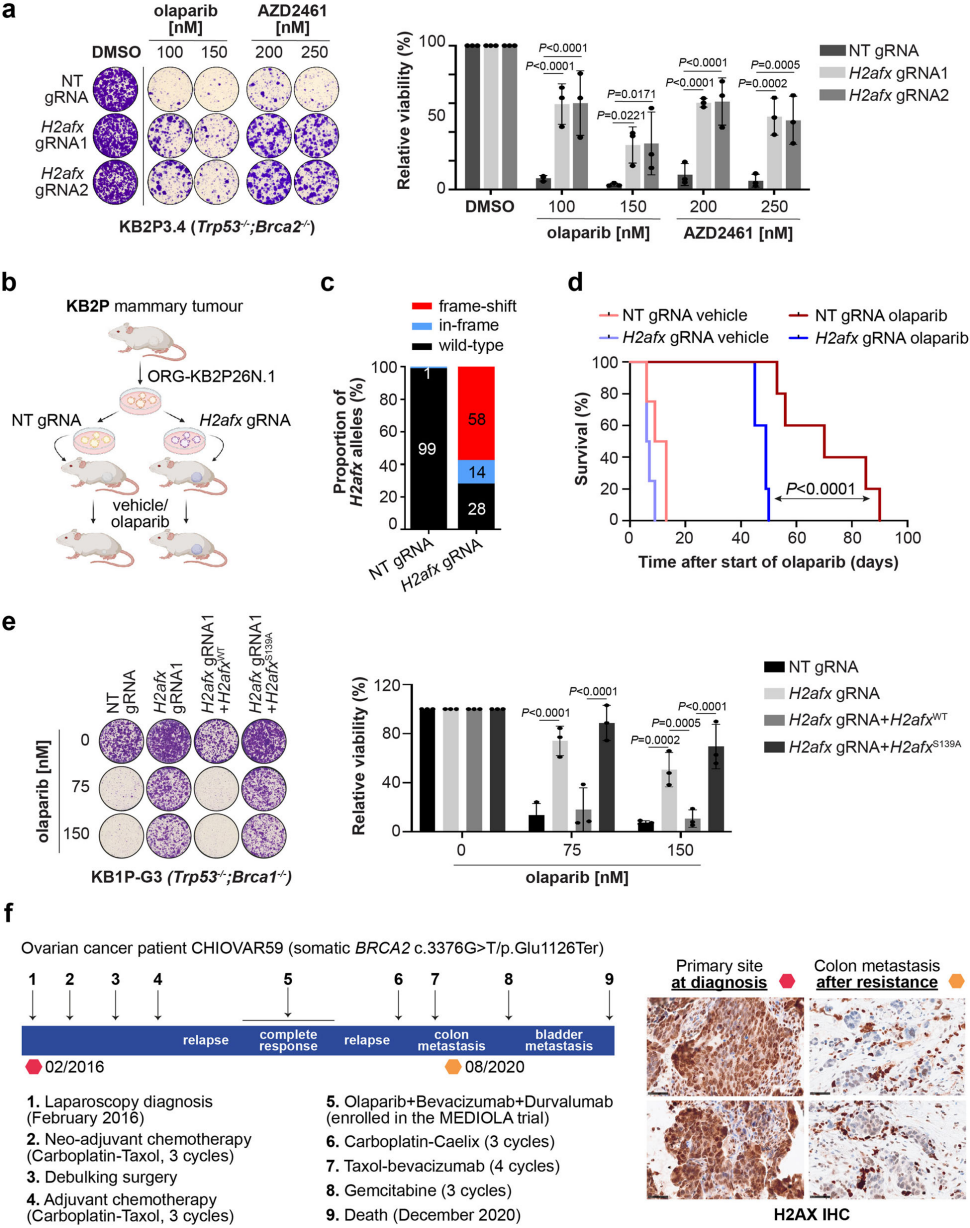
H2AX depletion leads to PARPi resistance in vitro and in vivo. **a** Clonogenic survival assay of KB2P3.4-derived cells treated, or mock treated, with the indicated concentrations of the PARPi olaparib and AZD2461 for 12 days. Plotted values express the mean ± SD of clonogenic survival (*n* = 3 independent experiments). *P*-values were calculated with two-way Anova test and adjusted for multiple comparisons. Source data are provided as a Source Data file. **b** Schematic design of the in vivo experiment. **c** Allelic modification rate of H2AX-deficient ORG-KB2P26N.1 organoids evaluated by TIDE analysis prior to transplantation. **d** Kaplan-Meier curves showing the survival of vehicle- or olaparib-treated mice bearing H2AX-proficient or deficient KB2P tumours. Each group contained 5 animals. *P*-value was calculated with the Mantel-Cox test. Source data are provided as a Source Data file. **e** Clonogenic survival assay of KB1P-G3-derived cells expressing the indicated H2AX variants and treated as in (**a**). Plotted values express the mean ± SD of clonogenic survival (*n* = 3 independent experiments). *P*-values were calculated with two-way Anova test and adjusted for multiple comparisons. Source data are provided as a Source Data file. **f** Therapy history of the CHIOVAR59 patient. On the right, H2AX IHC was performed on two tissue biopsies, primary tumour at diagnosis and colon metastasis after therapy resistance. Two areas of the slide are shown for each biopsy. Scale bar is 50 µm.

To validate our finding, we then depleted *H2afx* by CRISPR-Cas9 in KB2P3.4 (*Trp53*^*-/-*^*;Brca2*^*-/-*^) and KB1P-G3 (*Trp53*^*-/-*^*;Brca1*^*-/-*^) cells and studied PARPi response in vitro (Supplementary Fig. 1a-d). Consistent with our previous genetic analysis, *H2afx* depletion rescued the cellular sensitivity to the PARP inhibitors olaparib, AZD2461, and talazoparib in both KB2P3.4 and KB1P-G3 cells (Fig. 2a; Supplementary Fig. 1e, f). We also examined the impact of *H2afx* status on tumour growth in vivo^27^ (Fig. 2b). For this purpose, we transduced our BRCA2-deficient organoid line ORG-KB2P26N.1 with a non-targeting (NT) or a *H2afx* gRNA and verified the frameshift mutation rate by TIDE analysis (Fig. 2c). Next, we injected the modified organoids orthotopically into the inguinal mammary fat pad of female nude mice and waited until the tumour reached a palpable size (50–100 mm^3^). Mice were then randomized and 100 mg olaparib per kg or vehicle was administered daily by intraperitoneal injection. Strikingly, mice with *H2afx*-depleted tumours responded worse to the olaparib treatment than the *H2afx* wildtype counterparts and had to be sacrificed earlier (Fig. 2d; Supplementary Fig. 2e). We also generated stable *H2AFX*^*-/-*^ clones in human RPE1*-*h*TERT TP53*^*-/-*^*;BRCA1*^*-/-*^ cells and all showed full resistance to olaparib (Supplementary Fig. 2a). Moreover, shRNA-mediated depletion of *H2AX* caused olaparib resistance in the triple-negative breast cancer cell line MDA-MB-436, that carries the *BRCA1 c.5277+1* *G* > *A* mutation and shows complete loss of BRCA1 protein product^28^ (Supplementary Fig. 2b, c). The H2AX-dependent PARPi sensitivity fully depended on Serine 139 phosphorylation, as we could fully complement PARPi response in KB1P-G3 cells with a wild-type H2AX but not with the H2AX^S139A^ mutant (Fig. 2e; Supplementary Fig. 2d). Moreover, we also noticed that H2AX-depleted cells had reduced cellular sensitivity to the crosslinking agent cisplatin but not to ionizing radiation (IR) (Supplementary Fig. 1g, h), indicating that the mechanism of resistance is likely not associated with general DNA damage signalling.

Based on these results we tested H2AX expression by immunohistochemistry using paired biopsies that we obtained from a high-grade serous ovarian carcinoma patient carrying a pathogenic somatic mutation in *BRCA2* (c.*3376* *G* > *T*/ p.Glu1126Ter) (Fig. 2f). The CHIOVAR59 patient received neo- and adjuvant platinum-based therapy. At first relapse, she was treated with olaparib combined with anti-VEGF and anti-PD-L1 (Fig. 2f), resulting in complete response. Fifteen months later, the patient had a second relapse with a metastasized peritoneal tumour resistant to all subsequent therapies. Compared to the sample taken before chemotherapy, we found a clear H2AX reduction in tumour cells of the drug-resistant colon metastasis (Fig. 2f). Although anecdotal, this case indicates that H2AX loss is a clinically relevant mechanism associated with chemotherapy resistance in BRCA-deficient tumours.

### H2AX loss restores replication fork protection in BRCA-deficient tumour cells

**Fig. 3:**
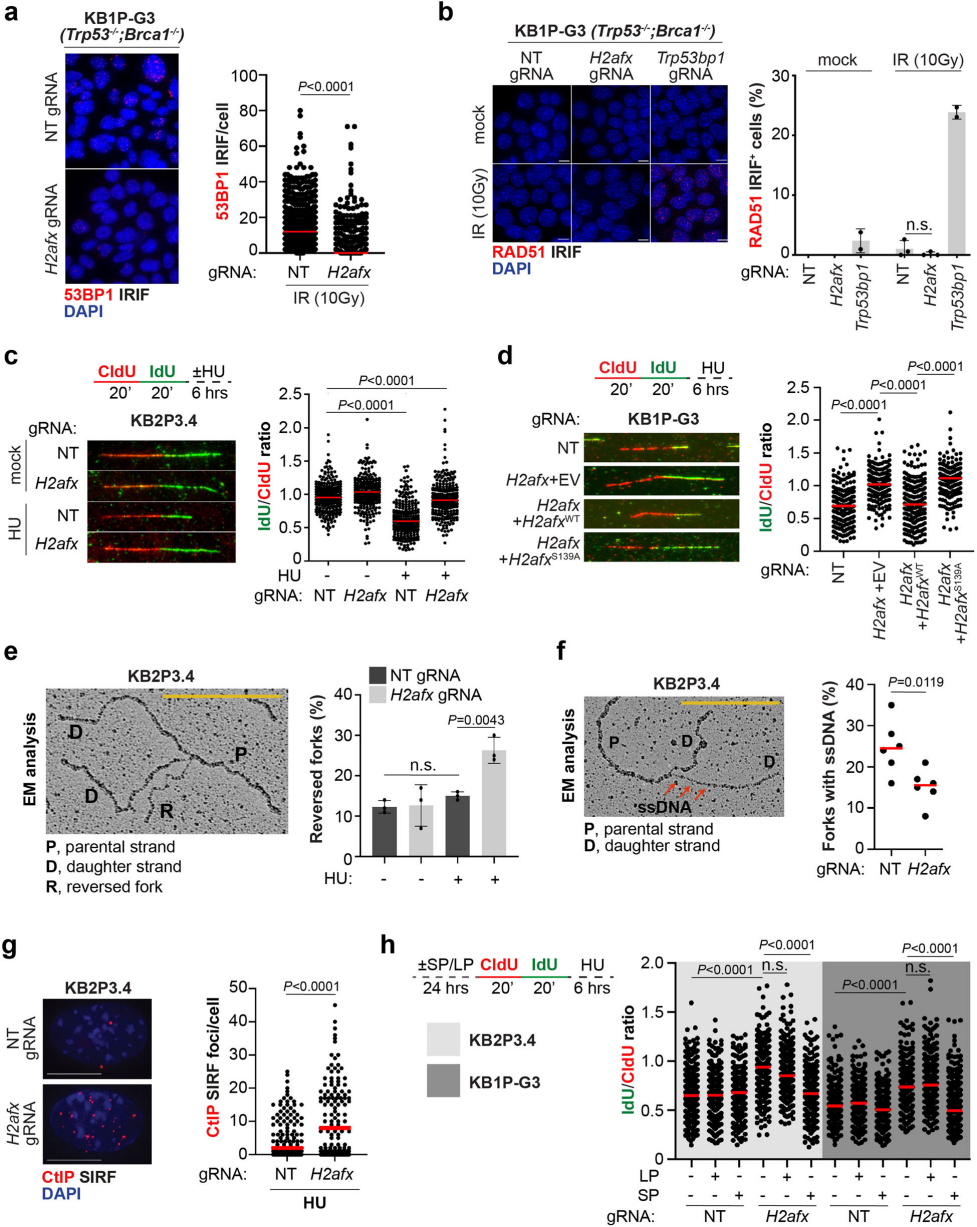
H2AX depletion restores replication fork protection. **a** 53BP1 Ionizing Radiation-Induced Foci (IRIF) analysis in KB1P-G3 cells 4 h after 10 Gy exposure. Plotted values show the median of 53BP1 IRIF/cell from at least 600 cells (*n* = 2 independent experiments). *P*-values were calculated with one-way Anova test. Source data are provided as a Source Data file. **b** RAD51 IRIF analysis in KB1P-G3 cells treated as in (**a**). The presented data are the mean ± SD (*n* = 2 and *n* = 3 independent experiments for 53bp1-depleted and the other cell lines, respectively). Source data are provided as a Source Data file. **c** DNA fiber analysis in KB2P3.4 cells treated according to the depicted scheme. Hydroxyurea (HU) was used at 8 mM for 6 h. Plotted values show the median of individual IdU/CldU ratios from at least 200 fibers (*n* = 3 independent experiments). *P*-values were calculated with one-way Anova test and adjusted for multiple comparisons. Source data are provided as a Source Data file. **d** DNA fiber analysis in the indicated KB1P-G3 cells treated as in (**c**). Plotted values show the median of individual IdU/CldU ratios from at least 300 fibers (*n* = 3 independent experiments). *P*-values were calculated with two-way Anova test and adjusted for multiple comparisons. Source data are provided as a Source Data file. **e** Electron Microscopy (EM) analysis of reversed fork intermediates following HU treatment as in (**c**). The electron micrograph represents a reversed replication fork. P parental strand, D daughter strand, R regressed arm. The presented data are the mean ± SD (*n* = 3 independent experiments). *P*-values were calculated with the unpaired two-tailed Student’s *t*-test. Source data are provided as a Source Data file. **f** EM analysis showing the median number of forks with detectable ssDNA at the junction in cells treated as in (**e**). *P*-value was calculated with the unpaired two-tailed Student’s *t*-test. Source data are provided as a Source Data file. **g** CtIP SIRF in KB2P3.4-derived cells treated as in (**c**). Plotted values show the median of CtIP SIRF foci/cell from at least 140 cells (*n* = 3 independent experiments). *P*-values were calculated with one-way Anova test. Source data are provided as a Source Data file. **h** KB2P3.4 and KB1P-G3-derived cells were pretreated for 24 h with the indicated CtIP peptides prior to analog in vivo labeling according to the depicted scheme. HU was used at 8 mM and the CtIP peptides were used at 10 µM. Plotted values show the median of individual IdU/CldU ratios from at least 250 fibers (*n* = 2 independent experiments). *P*-values were calculated with one-way Anova test and adjusted for multiple comparisons. Source data are provided as a Source Data file.

We then dissected the molecular mechanism how H2AX loss promotes chemotherapy resistance. *H2afx*-depleted cells had reduced levels of micronuclei after olaparib treatment (Supplementary Fig. 3a), indicating reduced levels of genomic instability. Reduction in micronuclei formation is usually associated with the restoration of conservative HR repair, particularly in BRCA1-deficient cells^29^. Given the role of γH2AX in stabilizing 53BP1 near DNA lesions^30,31^, we tested whether H2AX loss increases PARPi resistance by preventing 53BP1 complex formation and restoring competent HR. Consistent with the function of γH2AX in recruiting 53BP1 near DNA damage sites, we observed that 53BP1 IRIF were significantly reduced in KB1P-G3 *H2afx*-depleted cells (Fig. 3a). Nevertheless, in contrast with 53BP1-deficient cells, *H2afx* depletion failed to restore RAD51 IRIF (Fig. 3b). This stark difference in RAD51 IRIF formation also reflected a different cellular response to olaparib, which was stronger in 53BP1-depleted cells as shown by their increased cell survival and the increased IC_50_ values after olaparib treatment (Supplementary Fig. 3b, c). However, to our surprise, we noticed that H2AX-deficient cells had increase cellular resistance to the replication fork stalling agent hydroxyurea (HU), which instead was not observed in 53BP1-depleted cells (Supplementary Fig. 3d). These results confirm that H2AX and 53BP1 control drug resistance via two genetically separable mechanisms (Supplementary Fig. 3e). We therefore inferred that H2AX loss may increase chemotherapy resistance via another proposed mechanism, that is the restoration of replication fork protection^20^. Consistent with this hypothesis, single molecule analysis of replication tracts showed that, in marked contrast with BRCA1/2-deficient cells where stalled forks undergo extensive nucleolytic degradation^32–37^, H2AX loss restored fork integrity in both KB1P-G3 and KB2P3.4 cells in response to hydroxyurea and camptothecin (Fig. 3c, d; Supplementary Fig. 4a). Importantly, a similar phenotype was not observed in 53BP1-deficient cells, where stalled forks still undergo extensive nucleolytic degradation (Supplementary Fig. 4b). Importantly, H2AX loss did not alter normal fork elongation rates (Supplementary Fig. 3f) which we recently observed in *Mdc1*-deleted cells^38^. H2AX loss also restored replication fork stability in the BRCA1-deficient MDA-MB-436 cell line (Supplementary Fig. 4c, d). Consistent with the critical role of H2AX Serine 139 phosphorylation for PARPi resistance (Fig. 2c), fork degradation was entirely abolished in KB1P-G3 H2AX-deficient cells complemented with the H2ax^S139A^ variant (Fig. 3d), indicating that H2AX phosphorylation on Serine 139 plays a critical role at stressed replication forks in BRCA-deficient cells.

The reduced fork resection observed in H2AX-deficient cells may be explained by a defect in fork reversal^34–38^, or by an increased protection of the regressed arms^19,20^. To understand which of the fork transaction steps is controlled by H2AX, we directly looked at fork architecture by electron microscopy (EM) analysis^39^. Notably, we reproduced the lack of reversed fork intermediates in BRCA2-deficient cells treated with HU (Fig. 3e), which has been previously attributed to the unrestrained nuclease activity at stalled forks^34–37^. In contrast, KB2P3.4 *H2afx*-depleted cells showed a significantly higher number of reversed fork intermediates after HU treatment (Fig. 3e), indicating that H2AX is not involved in the initial reversal step but rather in the protection of the newly formed regressed intermediates. Our EM analysis also revealed fewer replication intermediates with detectable single stranded DNA (ssDNA) at the fork junction in KB2P3.4 *H2afx*-deleted cells (Fig. 3f), likely indicating a less pronounced degradation from the reversed fork end. Suppression of ssDNA gaps, another recently proposed mechanism of PARPi tolerance^40–42^, was also assessed using the DNA fiber assay followed by in vitro S1 nuclease digestion^43^. This approach confirmed that the IdU/CldU ratio in H2AX-deficient cells is not altered (Supplementary Fig. 4e), corroborating our EM findings. Notably, these short ssDNA patches observed at the fork junction differ from the recently described post replicative gaps, as these intermediates were not detected far away from the fork, which would be indicative of aberrant Okazaki fragment processing in H2AX-deficient cells.

MRE11 has been proposed to be the main nuclease active at stalled forks, particularly in BRCA2-deficient cells^32–37,44^. However, we did not detect any major reduction in the MRE11 recruitment on nascent DNA after replication stress in KB2P3.4 *H2afx*-deleted cells by in situ analysis of protein interactions at DNA replication forks (SIRF)^45^ (Supplementary Fig. 4g). Given these results, we tested whether H2AX loss could increase replication fork protection without affecting nuclease dynamics. During the G_0_/G_1_ phase of the cell cycle, H2AX has been shown to inhibit the activity of CtIP^46^, a protein with crucial roles in DNA end resection but also with a key role in replication fork protection^47,48^. Therefore, we examined whether H2AX may similarly counteract CtIP-mediated fork protection. In agreement with previous findings^46^, we observed a stronger association of CtIP with stalled forks in KB2P3.4 and MDA-MB-436 H2AX-depleted cells (Fig. 3g; Supplementary Fig. 4f). To verify whether the increased association of CtIP at stalled forks coincides with increased fork protection, we monitored fork stability in BRCA-deficient *H2afx*-deleted cells depleted for CtIP. Since stable CtIP depletion significantly affects the proliferation of BRCA1/2-deficient cells^48^, we transiently treated KB2P3.4 and KB1P-G3 H2AFX-depleted cells with a hydrocarbon-stapled peptide (SP) that specifically interferes with CtIP functions by targeting its tetramerization^49^. Strikingly, pretreatment with the CtIP-SP, but not with a linear peptide (LP), restored fork degradation in both KB2P3.4 and KB1P-G3 H2AX-depleted cells (Fig. 3h). We infer from these data that the effect of H2AX loss in PARPi resistance in BRCA-deficient cells is mediated by the increased association of CtIP with stalled forks. Consistently, treatment with a partial inhibitory concentration of the CtIP-SP of 5 µM resensitised KB1P-G3 H2AX-depleted cells to PARPi (Supplementary Fig. 4h).

### Sensitivity to ATM inhibitors is an acquired vulnerability of H2AX-deficient tumours

**Fig. 4:**
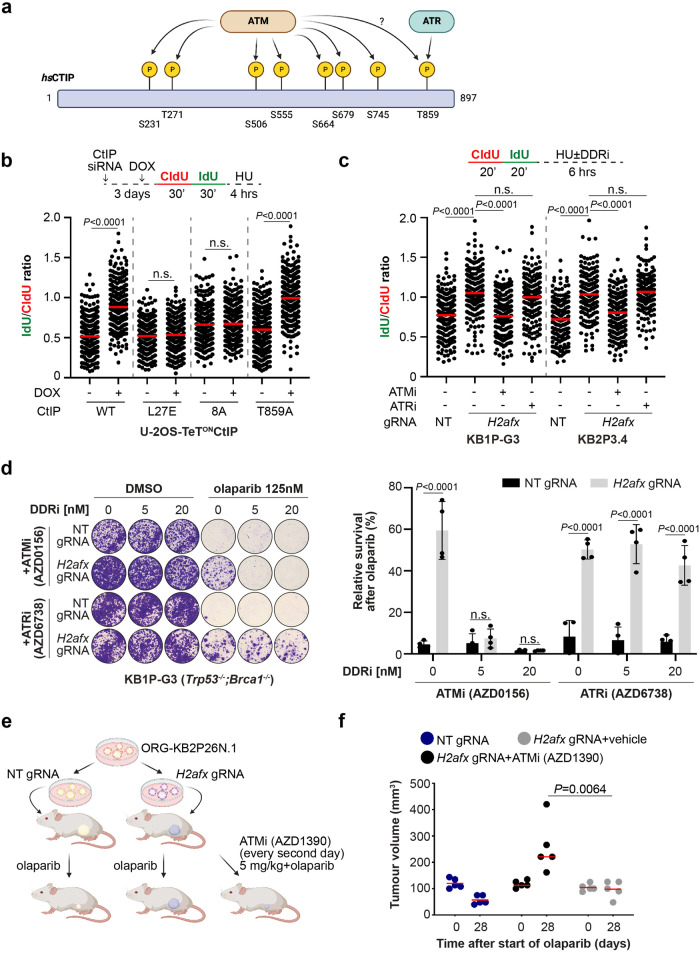
ATM inhibition restores PARPi sensitivity in H2AX-deficient mammary tumours. **a** Human CtIP protein map with highlighted the 8 ATM and ATR phosphorylation sites. **b** DNA fiber analysis in U-2OS-derived cells transfected with CtIP siRNA and treated according to the depicted scheme. 24 h before the experiment, CtIP^WT^, CtIP^L27E^, CtIP^8A^ or CtIP^T859A^ expression was induced by doxycycline treatment. Plotted values show the median of individual IdU/CldU ratios from at least 300 fibers (*n* = 2 independent experiments). *P*-values were calculated with one-way Anova test and adjusted for multiple comparisons. Source data are provided as a Source Data file. **c** DNA fiber analysis in KB1P-G3 and KB2P3.4-derived cells treated according to the depicted scheme. HU was used at 8 mM, AZD0156 and AZD6738 were used at 10 µM. Plotted values show the median of individual IdU/CldU ratios from at least 170 fibers (*n* = 3 independent experiments). *P*-values were calculated with one-way Anova test and adjusted for multiple comparisons. Source data are provided as a Source Data file. **d** Clonogenic survival assay of KB1P-G3-derived cells treated, or mock treated, with the indicated concentrations of olaparib, AZD0156, and AZD6738 for 12 days. Plotted values express the mean ± SD of clonogenic survival (*n* = 4 independent experiments). *P*-values were calculated with two-way Anova test and adjusted for multiple comparisons. Source data are provided as a Source Data file. **e** Schematic design of the in vivo experiment with the ATMi AZD1390. **f** Plotted values express the median size (mm^3^) of individual tumours from five animals transplanted with the indicated organoid lines and subjected to the indicated treatment for 28 consecutive days after tumour formation. *P*-values were calculated with two-way Anova test corrected with the Bartlett’s test. Source data are provided as a Source Data file.

The DNA Damage Response (DDR) kinases ATM and ATR are known to phosphorylate CtIP at several conserved S/T-Q residues (Fig. 4a)^50–52^. ATM-, but not ATR-, mediated phosphorylation stimulates CtIP endonuclease activity in vitro^51^, a function which has latter been shown to dampen nucleolytic degradation of stalled forks^48^. We asked whether CtIP phosphorylation by DDR kinases is required for replication fork protection. To test this, we generated doxycycline-inducible U-2OS lines that express a siRNA-resistant CtIP^WT^, a CtIP^8A^ variant where all its S/T-Q sites were mutated to alanine, a CtIP^T859A^ variant with a point mutation only in the ATR phosphorylation site, and a CtIP^L27E^ variant entirely lacking its tetramerization capacity^51^ (Supplementary Fig. 5a). Strikingly, CtIP^WT^ expression complemented the lack of fork protection elicited by endogenous CtIP depletion, while the CtIP^8A^ variant failed to complement the loss of endogenous CtIP in a similar fashion to the catalytically inactive CtIP^L27E^ mutant (Fig. 4b). Interestingly, the CtIP^T859A^ mutant is proficient in fork protection (Fig. 4b), consistent with previous results showing intact nuclease activity of CtIP-T859A mutant. Next, we asked whether these results could be recapitulated by using the clinically relevant ATMi AZD0156 and the ATRi AZD6738. Remarkably, the ATMi AZD0156, but not the ATRi AZD6738, robustly restored fork degradation in BRCA- H2AX-deficient cells (Fig. 4c), indicating that the ATR signalling branch is dispensable for fork protection in these tumours. We also observed loss of CtIP localization at stalled forks by SIRF upon AZD0156 treatment (Supplementary Fig. 5b), suggesting that ATM controls CtIP function at stalled forks by promoting its catalytic activity and its proper localization. Next, we investigated the effects of the same DDRi on PARPi resistance. Consistent with the above results, and in agreement with the idea that fork stabilization is the underlying mechanism of resistance in BRCA- H2AX-deficient tumours, partially inhibitory ATMi concentrations (AZD0156, 5-20 nM) entirely rewired PARPi response in BRCA- H2AX-deficient cells (Fig. 4d; Supplementary Fig. 5c). In parallel, we also tested the same partially inhibitory concentrations of the ATRi AZD6738, which failed to restore PARPi sensitivity in these cells (Fig. 4d). Interestingly, we have found that *H2AFX* scored as one of the strongest hits in loss-of-function CRISPR-Cas9 screens performed in distinct human cancer cell lines treated with the ATMi AZD0156 (Supplementary Fig. 5d), as also previously found. Based on these results, we selected the hypothesis that the chemotherapy resensitization by the ATMi could be particularly important in tumours which have lost H2AX. In line with this idea, we found that AZD0156 failed to restore PARPi sensitivity in the chemoresistant KB2P1.21 *Mdc1*^-/-^ cells (Supplementary Fig. 5e), where PARPi resistance does not arise from restored replication fork protection (Supplementary Fig. 5f). These results strongly indicate that ATMi-induced resensitisation to chemotherapy is not due to the general inhibition of DNA repair transactions or due to the increase of replication stress levels, but rather on the specific deregulation of CtIP function at stressed forks. Finally, we explored whether ATMi restores PARPi sensitivity also in H2AX-deficient tumours in vivo (Fig. 4e). To address this question, we performed a similar experiment as shown in Fig. 2e and treated mice with the brain-penetrant ATMi AZD1390^53^ (5 mg/kg, every 2nd day) for the whole duration of the olaparib treatment (Fig. 4e). Again, we observed that in the animals with the *H2afx*-depleted organoids, tumours did not respond to the olaparib treatment and grew over the therapy course compared to the tumours in the control group (Fig. 4f). However, consistent with our in vitro data, the AZD1390 combination restored PARPi response and impaired tumour growth throughout the duration of the experiment (Fig. 4f). Together, these data indicate that ATM inhibitors in combination with PARPi can be effectively used to overcome drug resistance in H2AX-deficient tumours and control tumour growth.

## Discussion

In this manuscript, we have shown a role for the histone H2AX in replication fork biology in BRCA1/2-deficient tumours. Previous work in BRCA-proficient cells reported a function for the histone H2AX in maintaining the stability of reversed replication forks^54^. However, we would like to emphasize that Schmid et al. ^54^. have analyzed the contribution of the H2AX-ATM checkpoint axis on fork slowing/reversal during unstressed replication, while we have focused on the role of H2AX-ATM in response to replication stress. This distinction is critical, as it aligns with emerging evidence suggesting that fork transactions may exhibit distinct genetic dependencies during unstressed replication compared to when exposed to genotoxic agents^38,55^. Thanks to the molecular characterization of the mechanisms behind the stabilization of stressed forks, we also identified an acquired genetic vulnerability of H2AX-deficient tumours, which is the inhibition of the ATM kinase. This discovery is of great interest and may have direct implications for the treatment of *BRCA*-mutated cancers at the metastatic stage, such as for the CHIOVAR59 case reported here. Future work on a larger scale will establish the clinical relevance of H2AX status for chemotherapy resistance and will help redirecting current therapeutic regimens towards more rationalized combination treatments.

## Methods

Our research complies with all the relevant ethical regulations defined by the Swiss Federal Office of Public Health, the Ethics Committee of the Canton of Geneva (CCER, Switzerland), the Animal Ethics Committees of The Netherlands Cancer Institute (Amsterdam, The Netherlands), and the Canton of Bern (Switzerland). All the experiments were performed in full compliance with national laws.

### Cell culture conditions

KB2P3.4 (*Trp53*^*-/-*^*;Brca2*^*-/-*^), KB2P1.21 (*Trp53*^*-/-*^*;Brca2*^*-/-*^), and KB1P-G3 (*Trp53*^*-/-*^*;Brca1*^*-/-*^) cells and all their derivative cell lines were grown at 37 °C in 3% O_2_ and cultured in Dulbecco’s modified Eagle medium Nutrient mixture F-12 (DMEM/F12; Gibco) supplemented with 10% Fetal Bovine Serum (FBS), pen/strep solution (50 U/ml), 5 ng/ml cholera toxin (Sigma Aldrich), insulin (5 µg/ml, Sigma Aldrich) and 5 ng/ml murine Epidermal Growth Factor (mEGF, Sigma Aldrich). ORG-KB2P17S.1 organoids were grown at 37 °C in normal oxygen embedded in Cultrex Reduced Growth Factor Basement Membrane Extract Type 2 (BME, Trevigen), seeded on 24-well suspension plates (Greiner Bio-One) and cultured in complete mouse mammary gland organoid medium: AsDMEM/F12 supplemented with 1 M HEPES (Sigma), GlutaMAX (Invitrogen), pen/strep (Gibco), B27 (Gibco), 125µM N-acetyl-L-cysteine (Sigma), 50 ng/ml murine epidermal growth factor (mEGF, Invitrogen). Human RPE1-h*TERT TP53*^*-/-*^, RPE1-h*TERT TP53*^*-/-*^*;BRCA1*^*-/-*^, and the RPE1-h*TERT TP53*^*-/-*^*;BRCA1*^*-/-*^;*H2AFX*^*-/-*^ clones were grown at 3% O_2_ in Dulbecco’s modified Eagle medium Nutrient mixture F-12 (DMEM/F12; Gibco) and penicillin/streptomycin solution (100 U/ml). MDA-MB-436 cells were grown in RPMI supplemented with 10% FBS, 1% non-essential aminoacids. SW62O cells were grown in L-15 medium (Leibovitz), 10% FBS, 1X glutamax. HT29 cells were grown in DMEM, 10% FBS, 1% non-essential aminoacids, 1% L-glutamine. BT549 cells were grown in RPMI-1640, 10% FBS, 0.023 U/ml insulin. U-2OS-TeT^ON^-eGFP-CtIP^WT^, U-2OS-TeT^ON^-eGFP-CtIP^L27E^, U-2OS-TeT^ON^-eGFP-CtIP^8A^, U-2OS-TeT^ON^-eGFP-CtIP^T859A^ were grown at 37 °C in DMEM supplemented with 10% FCS, 1% pen/strep, 5 µg/ml Blasticidin, 200 µg/ml Zeocin. CtIP expression was induced 24 h before the experiment with 1 µg/ml of doxycycline. Mission shRNA H2AX #1 (NM_002105), and H2AX #2 (NM_005657) were purchased from Sigma. All cell lines were authenticated by *Brca1/2*-specific PCR-based genotyping (mouse)^13,21^ and they were regularly tested for mycoplasma contamination (Mycoalert, Lonza).

### Drugs and reagents

The following chemical reagents were used throughout the study: AZD2461 (kindly provided by AstraZeneca), olaparib (kindly provided by AstraZeneca and Syncom (Groningen, The Netherlands)), talazoparib (Selleckchem; #S7048), cisplatin (Teva; #7680479980428), hydroxyurea (Sigma; #H8627), AZD0156 (kindly provided by AstraZeneca), AZD6738 (Selleckchem; #S7693), AZD1390 (kindly provided by AstraZeneca), Mirin (Sigma; #M9948), camptothecin (Selleckchem; #S1288), 5*-*Iodo-2’-deoxyuridine (IdU) (Sigma; #I7125), 5-Chloro-2’-deoxyuridine (CldU) (Sigma; #C6891), Ethynyl-2’-deoxyuridine (EdU) (Sigma; #A10044). LP and SP (Bachem; #4143690 and #4111111, respectively).

### Antibodies

The following primary antibodies were used: mouse anti-HA (clone 16B12, 1:1,000, #901533, Biolegend), mouse anti-γ-Tubulin (clone 6H3.1, 1:1,000, #5886, Cell Signalling), rabbit anti-GFP (1:1,000, #2555, Cell Signalling), rabbit anti-53BP1 (1:800, #A300-272A, Bethyl laboratories), rabbit anti-RAD51 (1:1,000, #70-012, Bioacademia), rat anti-BrdU (CldU) (clone [BU1/75 (ICR1)], 1:250, #ab6326, Abcam), mouse anti-BrdU (IdU) (1:40, #347580, BD Biosciences), rabbit anti-CtIP (1:500, #A300-488A, Bethyl Laboratories), mouse anti-Biotin (1:200, #200-002-211, Jackson Immuno Research), rabbit anti-MRE11 (1:500, a kind gift from Arnab Ray Chaudhuri^19^).

### Genome-wide CRISPR/Cas9 screens

The PARPi resistance screens were performed in the KB2P3.4 tumour cell line, which was previously established from a KB2P tumour^21^. Mouse GeCKO_V2 library, pool B (62,804 gRNAs targeting 20,628 genes (3 gRNAs/gene) including 1,000 non-targeting gRNAs), was stably introduced into the cells by lentiviral transduction at multiplicity of infection (MOI) of 1.5. Mouse GeCKO_V2 CRISPR knockout pooled library was a kind gift from Feng Zhang^22^. 6 independent transductions were carried out to obtain mutagenized cells for biological replicates of the PARPi resistance screen. To perform the genetic screen with a 100 x library coverage, 6 × 10^6^ mutagenized KB2P3.4 cells in each replicate were plated in 10 cm flasks, at low density (30,000 cells per flask) and grown in medium containing 200 nM AZD2461 for 3 weeks. The medium with the PARPi was refreshed twice a week. Cells were harvested before and after PARPi treatment for genomic DNA isolation. Subsequently, gRNA sequences were amplified from genomic DNA by two rounds of PCR amplification. Resulting PCR products were purified using MinElute PCR Purification Kit (Qiagen) and submitted for Illumina sequencing. Quality control was performed using R software (R Core Team, 2022) and package *edgeR*. Sequence alignment and enrichment analysis (day 0 vs PARPi-treated population) was carried out using the R package MAGeCKFlute^56^. Dataset of MAGeCK MLE analysis results of the CRISPR/Cas9 screen on RPE1-h*TERT* cells was extracted from the Supplementary Table 1 of Nordermeer et al. ^16^. For the CRISPR-Cas9 screens with the ATMi AZD0156, SW620, HT29, and BT549 cells were infected with lentiviral particles containing the whole-genome sgRNA library (Horizon Discovery), subjected to puromycin selection, and passaged to ensure loss of affected protein products. Puromycin-resistant cells were exposed to 10 nM ATMi (AZD0156) for 21 days (SW620, HT29) or 35 days (BT549), and remaining cell pools were isolated. Genomic DNA was extracted from these (QIAamp DNA Blood Maxi kit (Qiagen #51194)) and from parallel cell cultures treated in the absence of AZD0156, and DNA libraries were prepared and sequenced using an Illumina NextSeq next generation sequencing platform. Analysis of NGS data sets *e.g*. sgRNA abundance was achieved using Horizon Discovery’s data processing scripts, based on published analysis tool MAGeCK.

### DDR shRNA-based genetic screens

PARPi resistance shRNA screens were previously described^18^. Briefly, a shRNA library targeting a DNA Damage Response (DDR) gene set was built based on a gene list described before and the NCBI search (terms: “DNA repair”, “DNA damage response”, “DNA replication”, “telomere-associated genes”)^14^. The shRNA library was stably introduced into the tumour cell line KB2P3.4, which was subsequently selected with the PARPi AZD2461 or olaparib for 3 weeks. Genomic DNA was purified before and after treatment, amplified and sequenced as described above. Sequence alignment and analysis were performed using the MAGeCK software, MAGeCK-VISPR Maximum Likelihood Estimation (MLE) module, and the R package MAGeCKFlute^56^.

### Lentiviral transductions

Lentiviral stocks were generated by transient transfection of HEK293FT cells. On day 0, 6 × 10^6^ HEK293FT cells were seeded in 150 cm cell culture dishes and on the next day transiently transfected with lentiviral packaging plasmids and the pLentiCRISPRv2 vector containing the respective *H2afx*-targeting gRNA or a non-targeting gRNA using 2 x HBS (280 nM NaCl, 100 mM HEPES, 1.5 mM Na_2_HPO_4_, pH 7.22), 2.5 M CaCl_2_ and 0.1 x TE buffer (10 mM Tris pH 8.0, 1 mM EDTA pH 8.0, diluted 1:10 with dH_2_O). After 30 h, virus-containing supernatant was concentrated by ultracentrifugation at 20,000 rcf for 2 h in a SW40 rotor and the virus pellet was finally resuspended in 100 μl PBS. The virus titer was determined using a qPCR Lentivirus Titration Kit (#LV900, Applied Biological Materials). For lentiviral transduction, 150,000 target cells were seeded in 6-well plates. 24 h later, virus at the MOI of 50 was applied with 8 μg/ml Polybrene (Merck Millipore). Virus-containing medium was replaced with medium containing puromycin (3.5 μg/ml, Gibco) 24 h later. Puromycin selection was performed for 3 days; subsequently cells were expanded and frozen down at early passage. Tumour-derived organoids were transduced according to a previously established protocol^57^. The target sites modifications of the polyclonal cell pools were analyzed by TIDE analysis.

### Gene editing

For CRISPR/Cas9-mediated genome editing, KB2P3.4 and KB2P1.21 cells or KB2P17S.1 tumour-derived organoids were transduced with the pLentiCRISPRv2 vector encoding non-targeting gRNA, *H2afx*-targeting gRNA1 or *H2afx*-targeting gRNA2. The cells were then grown under puromycin (3 μg/ml) selection for 5 days. All constructs were verified with Sanger sequencing. For CRISPR/Cas9-mediated targeting of *H2afx* and *Mdc1* genes in KB1P-G3, KB2P3.4, and KB2P1.21 cells, non-targeting gRNA, *H2afx*, and *Mdc1*-targeting gRNAs were cloned into the pX330 vector (Addgene; #42230). Sanger sequencing-verified pX330 plasmids containing the correct sequences of gRNAs were transfected in cells using the TransIT-LT1 transfection reagent (#MIR 6604, Mirus) according to the manufacturer’s protocol. The cells were then grown under Puromycin (3 μg/ml) selection for 5 days. CRISPR gRNA sequences were chosen from the GeCKO_V2 library. The gRNA sequences were as follows: m*H2afx* gRNA1: 5’-TCGTACACTATGTCCGGACG-3’; m*H2afx* gRNA2: 5’-GGCGCCGGCGGTCGGCAAGA-3’; Non-Targeting (NT) gRNA: 5’-TGATTGGGGGTCGTTCGCCA-3’; h*H2AFX* gRNA1: 5’-GACAACAAGAAGACGCGAATC-3’; m*Mdc1* gRNA1: 5’-GGTGTGTGGCGAATGGACAA-3’. H2ax reconstitution was performed using the pOZ-N-FH (a kind gift from Dipanjan Chowdury). The *H2afx* coding sequence from *Mus musculus* was ordered from Eurofins and cloned into the pOZ-N-FH backbone adding the 1 x HA tag at the N terminus using the in-fusion HD cloning kit (#12141, Takara). Full length wild type *H2afx* coding sequence was then mutagenized to obtain the desired S139A point mutation.

### gDNA isolation, amplification, and TIDE analysis

To assess the modification rate at the gRNA-targeted region of *H2afx*, cells were pelleted, and genomic DNA was extracted using the QIAmp DNA mini kit (Qiagen) according to manufacturer’s protocol. Target loci were amplified using Phusion High Fidelity Polymerase (ThermoFisher Scientific) using a 3-step protocol: 98 °C for 30”, 35 cycles at 95 °C for 15”, 55 °C for 15” and 72 °C for 30”, 72 °C for 7’. Reaction mix consisted of 10 µl of 2 x Phusion Mastermix (ThermoFisher Scientific), 1 µl of 10 µM forward (5’-CAATCACTGGGCGCGTTC-3’) and reverse (5’-TGGCTCAGCTCTTTCTGTGAG-3’) primers and 100 ng of DNA in 20 µl total volume. PCR products were purified using the QIAquick PCR purification kit (Qiagen) according to manufacturer’s protocol and submitted with corresponding sequencing primers for Sanger sequencing to confirm target modifications using the TIDE algorithm. The sequencing primers used for analysis of the modification rate in mouse cells are the following: mgRNA1 seq. primer: 5’-CAATCACTGGGCGCGTTC-3’; gRNA2 seq. primer: 5’-GAGTACCTCACTGCCGAG-3’; hgRNA1 seq. primer: 5’-GACAACAAGAAGACGCGAATC-3’.

### *H2afx* gene expression analysis in PARPi resistant mammary tumours

Differential *H2afx* gene expression analysis from normalized gene expression counts was evaluated using DIDS (version 0.10.1)^58^, selecting a threshold of *P* < 0.05 for statistical significance^12,18,58^.

### Clonogenic assays

To assess the growth and survival upon treatment with PARPi, Cisplatin, Hydroxyurea, or irradiation, KB2P3.4 and KB1P-G3 cells were seeded in 6-well plates in the following densities: 3000 cells/well (KB2P3.4) and 4,000 cells/well (KB1P-G3). The treatment of cells with DMSO or indicated concentrations of PARPi olaparib or AZD2461 started at the day of plating the cells and lasted for the whole duration of the experiment. The medium with DMSO or PARPi was refreshed twice a week. The control, DMSO-treated plates were fixed 7 days after seeding, the PARPi-treated plates were fixed after 12 days. For the cisplatin treatment, cells were plated 24 h prior addition of cisplatin-containing media. After 24 h, medium was refreshed, and all the plates were fixed after 7 days. Irradiation was carried out in a fractionated manner using the indicated irradiation doses 24, 48 and 72 h following plating of the cells. Plates with non-irradiated cells were fixed 7 days post plating, the irradiated cells were fixed after 10 days. The fixation was done with 4% formalin and the surviving colonies stained with 0.1% crystal violet. The cell survival and growth were analyzed in an automated manner using the ImageJ ColonyArea plugin. For the competition assays, cells were collected before and after the experiment for gDNA isolation and TIDE analysis as described above. For clonogenic assays with the DDRi, the same experimental setup as in the clonogenic assays with PARPi was used. DDRi were added to the medium on the day of plating and the medium containing the drug was refreshed twice/week until the end of the experiment.

### In vivo studies

All animal experiments were approved by the Animal Ethics Committee of the Netherlands Cancer Institute (Amsterdam, The Netherlands, license AVD30100202011584) and the Canton of Bern (Switzerland, license BE69/2021) and were performed in full compliance with national laws, which enforce Dir. 2010/63/EU (Directive 2010/63/EU of the European Parliament and of the Council of 22 September 2010 on the protection of animals used for scientific purposes). For tumour organoid transplantation, ORG-KB2P26N.1 organoids were collected, incubated with TripLE at 37 °C for 5’, dissociated into single cells, washed in PBS, resuspended in tumour organoid medium, and mixed in a 1:1 ratio of tumour organoid suspension and BME. Organoid suspension containing a total of 10^5^ cells were injected in the fourth right mammary fat pad of 6–9 week-old female NMRI nude mice. Tumour size was measured by caliper and tumour volume was calculated ((length x width^2^)/2). Treatment of tumour bearing mice was initiated when tumours reached a detectable size of at least ~ 25–50 mm^3^, at which point mice were separated into a vehicle-treated group (NT gRNA *n* = 5, *H2afx*-targeting gRNA1 *n* = 5) and an olaparib-treated group (NT gRNA *n* = 5, *H2afx*-targeting gRNA *n* = 5). Olaparib (100 mg/kg) was administered orally for 28 consecutive days. The control tumour-bearing mice were dosed with vehicle following the same the schedule as the PARPi group. Animals were anesthetized with isoflurane, sacrificed with CO_2_ followed by cervical dislocation when tumours reached a volume of ~ 1000 mm^3^. Tumour sampling included cryopreserved tumour pieces, fresh frozen tissue, and formalin-fixed material (4% (v/v) formaldehyde in PBS). The maximum body weight loss permitted is 10% of the total animal weight.

### H2AX immunohistochemistry on primary tumour samples

Patient CHIOVAR59 was enrolled in the biobank of ovarian cancer samples collected prospectively from ovarian cancer patients diagnosed at Hôpitaux Universitaires de Genève (HUG), Switzerland. CHIOVAR study was approved by the local ethics committee (CCER 2018-00407). The patient signed informed consent. Archival formalin-fixed paraffin embedded (FFPE) blocks were retrieved from the division of clinical pathology at HUG. Tumour and germline DNA was extracted and subjected to next-generation sequencing of a panel of 400 genes (NGS400v2 Agilent SureSelect XT HS) that revealed somatic mutation of *BRCA2* p.Glu1126Ter and *TP53* p.Ser166TyrfsTer4. Haematoxylin & Eosin sections from available FFPE blocks were reviewed by a board-certified pathologist (JCT). FFPE tissue sections were cut at 4 μm, dried overnight, dewaxed by immersion in xylene, rehydrated in ethanol of decreasing concentrations, subjected to heat-mediated antigen retrieval in pH6 citrate buffer for 10 min and incubated with a polyclonal anti-rabbit H2AX antibody (Novus Biologicals #NB100-383; 1:1000). H2AX immunohistochemistry was performed at the Translational Research Unit (University of Bern) with a BOND Research RX from Leica Biosystems as staining platform.

### Immunofluorescence

Cells were seeded on coverslips in 24-well plates 3 days prior the experiment. To analyze 53BP1 and RAD51 foci formation in H2AX-deficient KB1P-G3 cells, DSB were induced by γ-irradiation (10 Gy) 4 h prior to fixation. Subsequently, cells were washed in PBS and fixed with 4% (v/v) PFA/PBS for 20’ at RT. Fixed cells were washed with PBS and permeabilized for 20’ in 0.2% (v/v) Triton X-100/PBS. Next, slides were washed three times with 0.2% Tween-20/PBS and blocked with staining buffer (PBS, BSA (2% w/v), glycine (0.15% w/v), Triton X-100 (0.1% v/v)) for 1 h at RT. Incubation with the primary rabbit polyclonal anti-53BP1 (#A300-272A, Bethyl Laboratories) and anti-RAD51 antibody (#70-012, Bioacademia) diluted 1:1000 in staining buffer was carried out for 2 h at RT. Slides were then washed four times for 5’ with 0.2% (v/v) PBS-Tween-20 and then incubated with Goat anti-rabbit IgG (H + L) Cross-Absorbed Secondary Antibody, Texas Red-X (# T-6391, ThermoFisher Scientific) diluted 1:2000 in staining buffer for 1 h at RT. Slides were washed three times for 5’ with 0.2% PBS-Tween-20, once with PBS and then mounted with Duolink In Situ mounting medium with DAPI (#DUO82040, Sigma Aldrich). Z-stack fluorescent images were acquired using the DeltaVision Elite widefield microscope (GE Healthcare Life Sciences). Multiple fields of view were imaged per sample with Olympus 100X/1.40, UPLS Apo, UIS2, 1-U2B836 objective and sCMOS camera at the resolution 2048 × 2048 pixels. Deconvolution of the acquired images was performed by the softWoRx DeltaVision software. Image analysis was performed using Fiji image processing package of ImageJ. Briefly, all nuclei were detected by the “analyze particles” command and all the foci per nucleus were counted with the “find maxima” command. Data were plotted with Prism software.

### Analysis of micronuclei formation

KB2P3.4 cells were seeded on coverslips in 24-well plates and treated with DMSO or indicated concentrations of olaparib 24 h later. After 48 h of treatment, cells were washed with PBS and fixed with 4% (v/v) PFA/PBS for 20’ in RT. Cells were then washed 3 times in 0.2% (v/v) PBS-Tween-20 and permeabilized for 20’ in 0.2% (v/v) Triton X-100/PBS. Subsequently, slides were washed 3 times with PBS, counterstained with DAPI (1:50,000 dilution, #D1306, Life Technologies) and washed 5 more times with PBS before mounting in Fluorescence mounting medium (#S3023, Dako). Z-stack images were acquired using the DeltaVision Elite widefield microscope (GE Healthcare Life Sciences). Multiple fields of view were imaged per sample with Olympus 100X/1.40, UPLS Apo, UIS2, 1-U2B836 objective and sCMOS camera. The frequency of micronuclei positive cells was analyzed manually in Fiji.

### Replication fork progression by DNA fiber analysis

Fork progression was measured as described previously^43^. Briefly, asynchronously growing subconfluent KB2P1.21 or KB2P3.4 cells were labeled with 25 μM thymidine analogue 5-chloro-2’-deoxyuridine (CIdU) (#C6891, Sigma-Aldrich) for 20’, washed three times with warm PBS and exposed to 250 μM of 5-iodo-2′-deoxyuridine (IdU) for 20’. All cells were collected by trypsinization and 2 μl of this cell suspension was then mixed with 8 μL of lysis buffer (200 mM Tris-HCl, pH 7.4, 50 mM EDTA, and 0.5% (v/v) SDS) on a positively charged microscope slide. After 9’ of incubation at RT, the slides were tilted at an ~ 30–45° angle to stretch the DNA fibers onto the slide. The resulting DNA spreads were air-dried, fixed in 3:1 methanol/acetic acid, and stored at 4 °C overnight. Next day, the DNA fibers were denatured by incubation in 2.5 M HCl for 1 h at RT, washed five times with PBS and blocked with 2% (w/v) BSA in 0.1% (v/v) PBST (PBS and Tween 20) for 40’ at RT while gently shaking. The newly replicated CldU and IdU tracks were stained for 3 h at RT using two different anti-BrdU antibodies recognizing CldU (#ab6326, Abcam) and IdU (#347580, BD Biosciences), respectively. After washing five times with PBS-T the slides were stained with goat the anti-mouse IgG (H + L) Cross-Adsorbed Secondary Antibody, Alexa Fluor 488 (#A-11029, ThermoFisher Scientific) diluted 1:600 in blocking buffer and with the Cy3 AffiniPure F(ab’)_2_ Fragment Donkey Anti-Rat IgG (H + L) antibody (#712-165-513, Jackson Immuno Research) diluted 1:150 in blocking buffer. Incubation with secondary antibodies was carried out for 1 h at RT in the dark. The slides were washed five times for 3’ in PBS-T, air-dried and mounted in Fluorescence mounting medium (#S3023, Dako). Fluorescent images were acquired using the DeltaVision Elite widefield microscope (GE Healthcare Life Sciences). Multiple fields of view from at least two slides (technical replicates) of each sample were imaged using the Olympus 60X/1.42, Plan Apo N, UIS2, 1-U2B933 objective and sCMOS camera at the resolution 2048 × 2048 pixels. To assess fork progression, the sum of individual CldU and IdU track lengths was measured using the segmented line tool in ImageJ software. Statistical analysis was carried out using Prism.

### Replication fork stability by DNA fiber analysis

CldU and IdU pulse-labeling of asynchronously growing KB2P3.4 and KB1P-G3 cells expressing NT or *H2afx*-targeting gRNA was performed as described above. After pulse-labeling with IdU and three washes with PBS, medium containing 8 mM hydroxyurea (HU) was added for 6 h. Cells were then washed and harvested by trypsinization, and then processed as described above. Replication fork stability was analyzed by measuring the ratio between CldU and IdU tracks in ImageJ.

### Detection of post-replicative ssDNA gaps by DNA fiber analysis

Detection of post-replicative ssDNA gaps was carried out as previously described^43^. In brief KB1P-G3 cells, were pulse labelled with 25 μM CldU (15’) followed by 250 μM IdU (45’) with or without 50 nM CPT. Cells were then harvested by trypsinization, washed with PBS and pre-extracted with CSK buffer (100 mM NaCl, 10 mM MOPS pH 7, 3 mM MgCl_2_, 300 mM sucrose and 0.5% Triton X-100 in water) for 10’ at RT. Next, isolated nuclei were harvested by centrifugation and incubated for 30’at 37 °C with S1 nuclease buffer containing or not 20 U/ml of S1 nuclease (#EN0321, Thermo Fisher Scientific). Nuclei were then pelleted, resuspended in PBS and spread onto microscope slides as previously described.

### Immunoblotting

Cells were lysed for 40’ in RIPA buffer supplemented with halt protease and phosphatase inhibitor cocktail (100x) (#78420, Thermo Fisher Scientific) while briefly vortexed every 10’. Lysates were then centrifuged at 12,000 rcf for 10’ at 4 °C and the supernatant was collected to determine protein concentration using Pierce BCA Protein Assay Kit (#23225, Thermo Fisher Scientific). Before loading, protein lysates were denatured at 95 °C for 5’ in 6x SDS sample buffer. Proteins were separated by SDS/PAGE in 10% gel before wet transfer to 0.45μm nitrocellulose membranes (GE Healthcare) and blocked in 5% dry milk powder in TBS-T (100 mM Tris, pH 7.5, 0.9% NaCl, 0.05% Tween-20). Membranes were incubated with the mouse monoclonal anti-HA (1:1,000, #901533, Biolegend) and anti-γ-Tubulin (1:1,000, #5886, Cell Signalling), anti-GFP (1:1,000, #2555, Cell Signalling) primary antibodies for 2 h at RT. After three 5’ washes in TBS-T, anti-rabbit or anti-mouse horseradish peroxidase (HRP)-linked secondary antibodies (1:5,000, Cell Signalling) were applied for 1 h at RT. Images were acquired using Vilber FUSION FX chemiluminescent imager.

### SIRF (in Situ analysis of protein Interactions at DNA Replication Forks)

SIRF assay was performed as previously reported^45^. Cells were seeded on coverslips and the following day they were pulsed with 25 μM EdU for 10’. After the EdU pulse, cells were initially pre-extracted with CSK buffer on ice for 5’ and then fixed with 3.7% Paraformaldehyde at RT for 10’. Coverslips were then washed with PBS and stored overnight at 4 °C. The following day cells were permeabilized in 0.2% Triton X-100 in PBS for 5’ and then the click reaction (100 mM Tris pH 8, 100 mM CuSO_4_, 2 mg/ml sodium-L-ascorbate, 10 mM biotin-azide) was performed for 90’ at 37 °C. Slides were then blocked for 1 h at 37 °C with blocking solution (PBS, BSA 2%, glycine 0.15%, Triton X-100 0.1%), followed by incubation with primary antibodies for 1 h at 37 °C (rabbit anti-CtIP 1:500, Bethyl Laboratories #A300-488A; mouse anti-Biotin 1:200, #200-002-211, Jackson Immuno Research; rabbit anti-MRE11 1:500, a kind gift from Arnab Ray Chaudhuri). After antibody incubation, coverslips were washed 2X with Buffer A for 5’ at RT (Duolink kit). Each coverslip was then incubated for 1 h at 37 °C with Duolink PLA probes (Thermo Fisher Scientific) diluted in blocking solution. After 2 washes with Buffer A for 5’ at RT, probes were ligated for 30’ at 37 °C and amplified by polymerase reaction for 100’ at 37 °C. Coverslips were then washed 2X with Buffer B for 5’ at RT (Duolink kit) and then mounted with DAPI on microscope slides. Images were acquired on multiple stacks using the DeltaVision Elite widefield microscope with a 60X objective. Deconvolution of the images was done using the softWoRx DeltaVision software. The number of foci in each cell was scored with ImageJ and the statistical analysis was performed using Prism.

### Transmission electron microscopy of replication intermediates

The procedure was performed as described previously with minor modifications^39^. A total of 2.5–5.0 × 10^6^ asynchronously growing KB2P3.4 cells expressing either NT or *H2afx*-targeting gRNA were treated with 8 mM hydroxyurea for 5 h, washed with PBS and then harvested by trypsinization and resuspended in 10 mL of cold PBS. DNA was cross-linked by exposing the living cells twice to 4,5′,8-trimethylpsoralen at a final concentration of 10 μg/mL followed by 3’ irradiation pulses with UV 365 nm monochromatic light (UV Stratalinker 1800, Agilent Technologies). The cells were then washed repeatedly with cold PBS and lysed with a cell lysis buffer (1.28 M sucrose, 40 mM Tris-Cl, pH 7.5, 20 mM MgCl_2_, and 4% (v/v) Triton X-100). The nuclei were then digested in a digestion buffer (800 mM guanidine-HCl, 30 mM Tris-HCl, pH 8.0, 30 mM EDTA, pH 8.0, 5% (v/v) Tween 20, and 0.5% (v/v) Triton X-100) supplemented with 1 mg/mL proteinase K at 50 °C for 2 h. Genomic DNA was extracted with a 24:1 Chloroform:Isoamyl alcohol mixture by phase separation (centrifugation at 8,000 rcf for 20’ at 4 °C) and precipitated by addition of equal amount of isopropanol to the aqueous phase, followed by another centrifugation step (8,000 rcf for 10’ at 4 °C). The obtained DNA pellet was washed once with 1 mL of 70% ethanol, air-dried at RT, and resuspended by overnight incubation in 200 μL TE (Tris-EDTA) buffer at RT. 12 μg of the extracted genomic DNA was digested for 5 h at 37 °C with 100U restriction enzyme *PvuI*I-HF (#R3151S, New England Biolabs). The digest was cleaned up using a silica bead DNA gel extraction kit (#K0513, Thermo Fisher Scientific). The benzyldimethylalkylammonium chloride (BAC) method was used for native spreading of the DNA on a water surface and then loading it on carbon-coated 400-mesh magnetic nickel grids. After the spreading procedure, the electron density of the DNA was increased by platinum coating with the platinum-carbon rotary shadowing technique using the MED 020 High Vacuum Evaporator (Bal-Tec). The grids were then scanned in a semi-automated fashion using a transmission electron microscope (FEI Thalos 120, LaB6 filament) at high tension ≤ 120 kV and pictures were acquired with a bottom mounted CMOS camera BM-Ceta (4000 ×4000 pixels). The images were processed with MAPS Version 3.14 (Thermo Fisher Scientific) and analyzed using MAPS Offline Viewer Version 3.14.11 (Thermo Fisher Scientific). The percentage of RFs containing ssDNA stretches was evaluated by manual scoring. Samples from mock-treated and HU-treated cells were pooled together for the analysis of ssDNA-containing RFs.

### Statistics & reproducibility

Statistical parameters including sample size, number of biological replicates, applied statistical tests and statistical significance are reported in the corresponding figure legends or materials and methods section. Power analysis (using a power of 0.8) was used to predetermine sample size for the mouse experiments shown in Figs. 2d and 4f. No data were excluded from the analyses. Except Figs. 1d, 1e, 2d, 4f, experiments were not randomized, and the investigators were not blinded to allocation during experiments and outcome assessment.

### Reporting summary

Further information on research design is available in the Nature Portfolio Reporting Summary linked to this article.

### Supplementary information

Supplementary Information

Reporting Summary

Peer Review File

### Source data

Source Data

## Data Availability

All data will be shared upon request by the lead contact with no restrictions. Source data are provided with this paper. Raw sequencing data of the genetic screens are available in European Nucleotide Archive (ENA) under accession number PRJEB75036. and PRJEB74933. Raw sequencing data of WE-seq and RNA-seq reported in this paper are available in European Nucleotide Archive (ENA) under accession number PRJEB61242. for KB1P(M) tumours and PRJEB61243. for KB2P tumours. The remaining data are available within the Article, Supplementary Information, or Source Data file. Source data are provided with this paper.
