# Supplementary material for: H2AX promotes replication fork degradation and chemosensitivity in BRCA-deficient tumours
Source: Nat Commun. 2024 May 24;15:4430. doi: 10.1038/s41467-024-48715-1 (PMC11126719; doi:10.1038/s41467-024-48715-1)

Supplementary figures

H2AX promotes replication fork degradation and chemosensitivity in BRCA-deficient tumours

Diego Dibitetto[1,2,12,13*], Martin Liptay[1,2,12], Francesca Vivalda[3], Hülya Dogan[1,2], Ewa Gogola[4,5], Martín G. Fernández[1,2], Alexandra Duarte[4,5], Jonas A. Schmid[3], Morgane Decollogny[1,2], Paola Francica[1,2], Sara Przetocka[3], Stephen T. Durant[6], Josep V. Forment[6], Ismar Klebic[1], Myriam Siffert[1], Roebi de Bruijn[4,5], Arne N. Kousholt[5,7], Nicole A. Marti[1,2], Martina Dettwiler[1], Claus S. Sørensen[7], Jean-Christophe Tille[8], Manuela Undurraga[9], Intidhar Labidi-Galy[10,11], Massimo Lopes[3], Alessandro A. Sartori[3], Jos Jonkers[4,5*] and Sven Rottenberg[1,2,4*]

[1]Institute of Animal Pathology, Vetsuisse Faculty, University of Bern, 3012 Bern, Switzerland
[2]Cancer Therapy Resistance Cluster and Bern Center for Precision Medicine, Department for Biomedical Research, University of Bern, 3012 Bern, Switzerland
[3] Institute of Molecular Cancer Research, University of Zürich, Switzerland
[4]Division of Molecular Pathology, The Netherlands Cancer Institute, 1066CX Amsterdam, The Netherlands
[5]Oncode Institute, Amsterdam, The Netherlands
[6]DDR Biology, Bioscience, Oncology R&D, AstraZeneca, Cambridge, CB4 0WG, UK
[7] Biotech Research and Innovation Centre, University of Copenhagen, 2200 N Copenhagen, Denmark
[8]Division of Clinical Pathology, Department of Diagnostics, Hôpitaux Universitaires de Genève, Geneva, Switzerland
[9]Division of Gynecology, Department of Pediatrics and Gynecology, Hôpitaux Universitaires de Genève, Geneva, Switzerland
[10]Faculty of Medicine, Department of Medicine and Center of Translational Research in Onco-Hematology, University of Geneva, Swiss Cancer Center Leman, Geneva, Switzerland
[11]Department of Oncology, Hôpitaux Universitaires de Genève, 4, Rue Gabrielle Perret-Gentil, Geneva, 1205, Switzerland
[12]These authors contributed equally: Diego Dibitetto and Martin Liptay
[13]Present address: Department of Experimental Oncology, Istituto di Ricerche Farmacologiche Mario Negri IRCCS, via Mario Negri 2, 20156 Milan, Italy
*e-mail: sven.rottenberg@unibe.ch, j.jonkers@nki.nl, or diego.dibitetto@marionegri.it

Supplementary Fig. 1

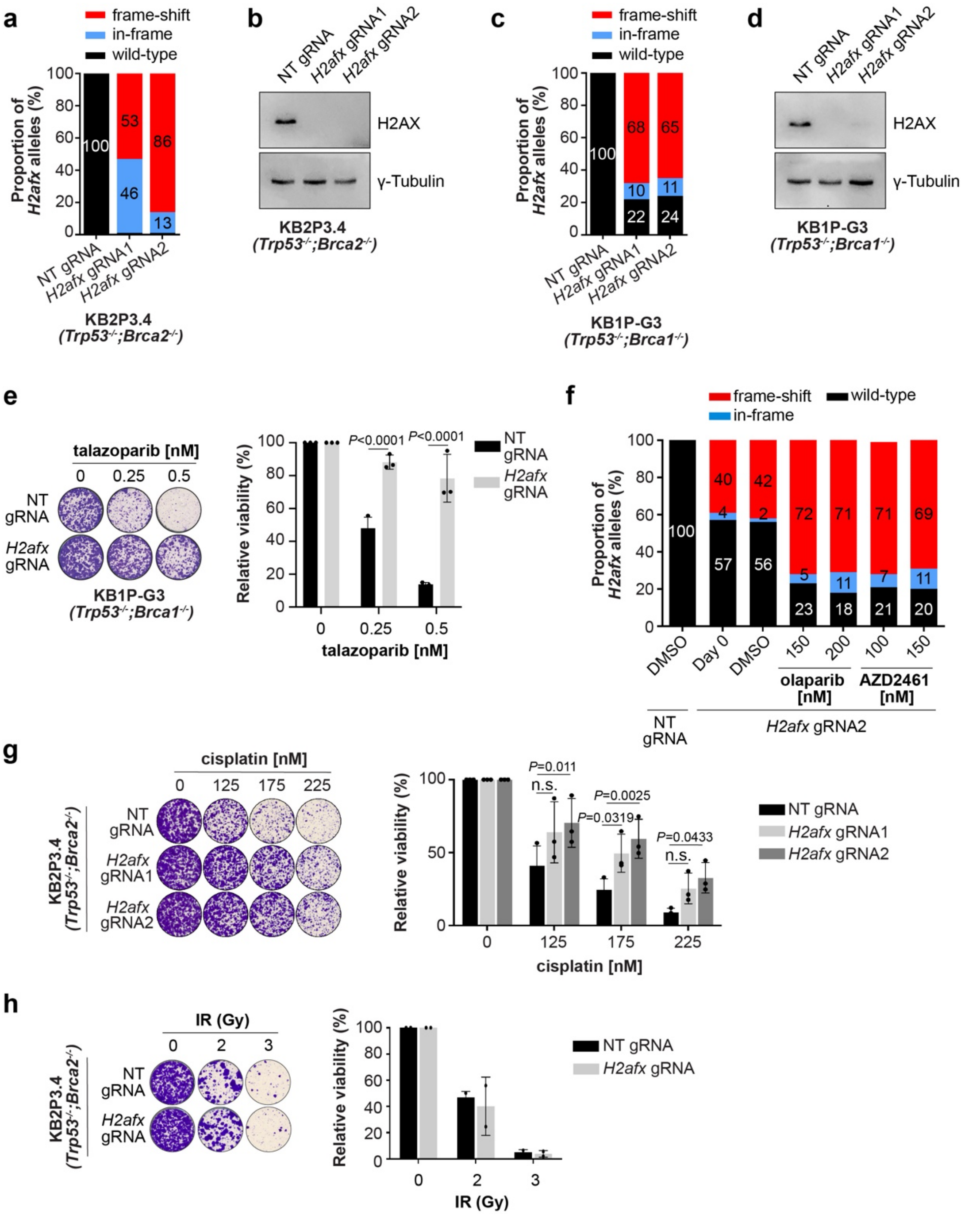

Supplementary Fig. 1. H2AX loss promotes resistance to PARPi and cisplatin but not ionizing radiation. a, *H2afx* allelic modification rate in KB2P3.4 cells evaluated by TIDE analysis. **b**, H2ax immunoblotting in KB2P3.4 cells. Source data are provided as a Source Data file. **c**, *H2afx* allelic modification rate in KB1P-G3 cells evaluated by TIDE analysis. **d**, H2AX immunoblotting in KB2P3.4 cells. Source data are provided as a Source Data file. **e**, Clonogenic survival assay of KB1P-G3-derived cells treated, or mock treated, with the indicated concentrations of talazoparib for 12 days. Plotted values are the mean±SD clonogenic survival (n=3 independent experiments). *P* values were calculated with two-way Anova test and

adjusted for multiple comparisons. **f**, *H2afx* allelic modification rate in a competition assay in KB2P1.21 cells evaluated by TIDE analysis. **g**, Clonogenic survival assay of KB2P3.4-derived cells treated, or mock treated, with the indicated concentrations of cisplatin for 12 days. Plotted values are the mean±SD clonogenic survival (n=3 independent experiments). *P* values were calculated with two-way Anova test and adjusted for multiple comparisons. **h**, Clonogenic survival assay of KB2P3.4-derived cells treated or not with the indicated IR doses and stained after 12 days. Plotted values are the mean±SD clonogenic survival (n=2 independent experiments).

Supplementary Fig. 2

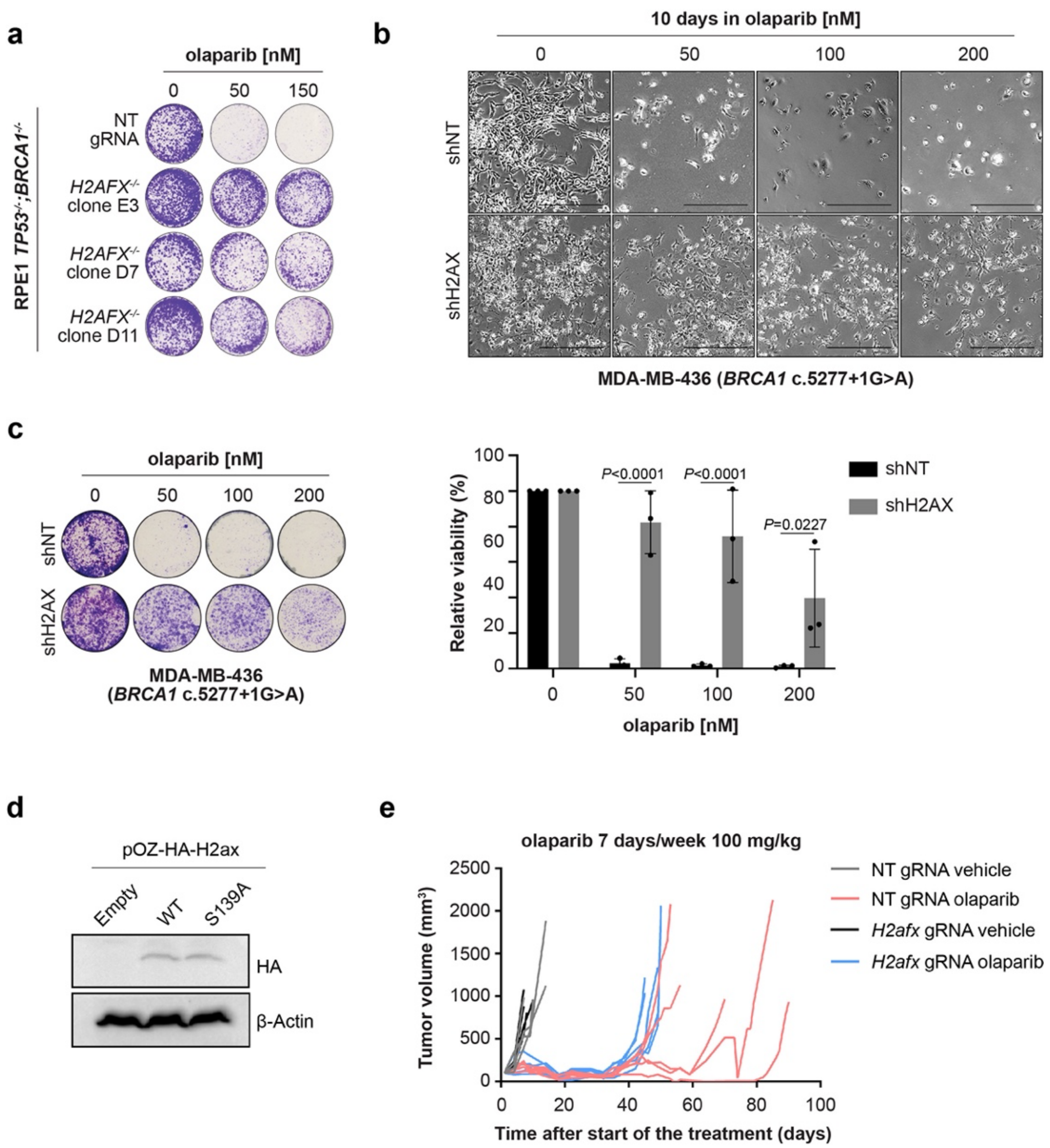

Supplementary Fig. 2. H2AX loss increases olaparib resistance in BRCA1-deficient human cells. a, Clonogenic survival assay of RPE1-h*TERT TP53*$^{-/-}$;*BRCA1*$^{-/-}$-derived cells treated, or mock treated, with the indicated concentrations of the PARPi olaparib for 12 days. **b**, Exemplificative pictures of MDA-MB-436-derived cells treated for 10 days with the indicated concentrations of olaparib. **c**, Clonogenic survival assay of MDA-MB-436-derived cells treated, or mock treated, with the indicated concentrations of olaparib for 15 days. Plotted values are the mean±SD clonogenic survival (n=3 independent experiments). *P* values were calculated with two-way Anova test and adjusted for multiple comparisons. **d**, H2ax immunoblotting in KB1P-G3 after genetic complementation. Source data are provided as a Source Data file. **e**, Each line represent the growth of an individual tumour from the *in vivo* experiment shown in Fig. 2d.

Supplementary Fig. 3

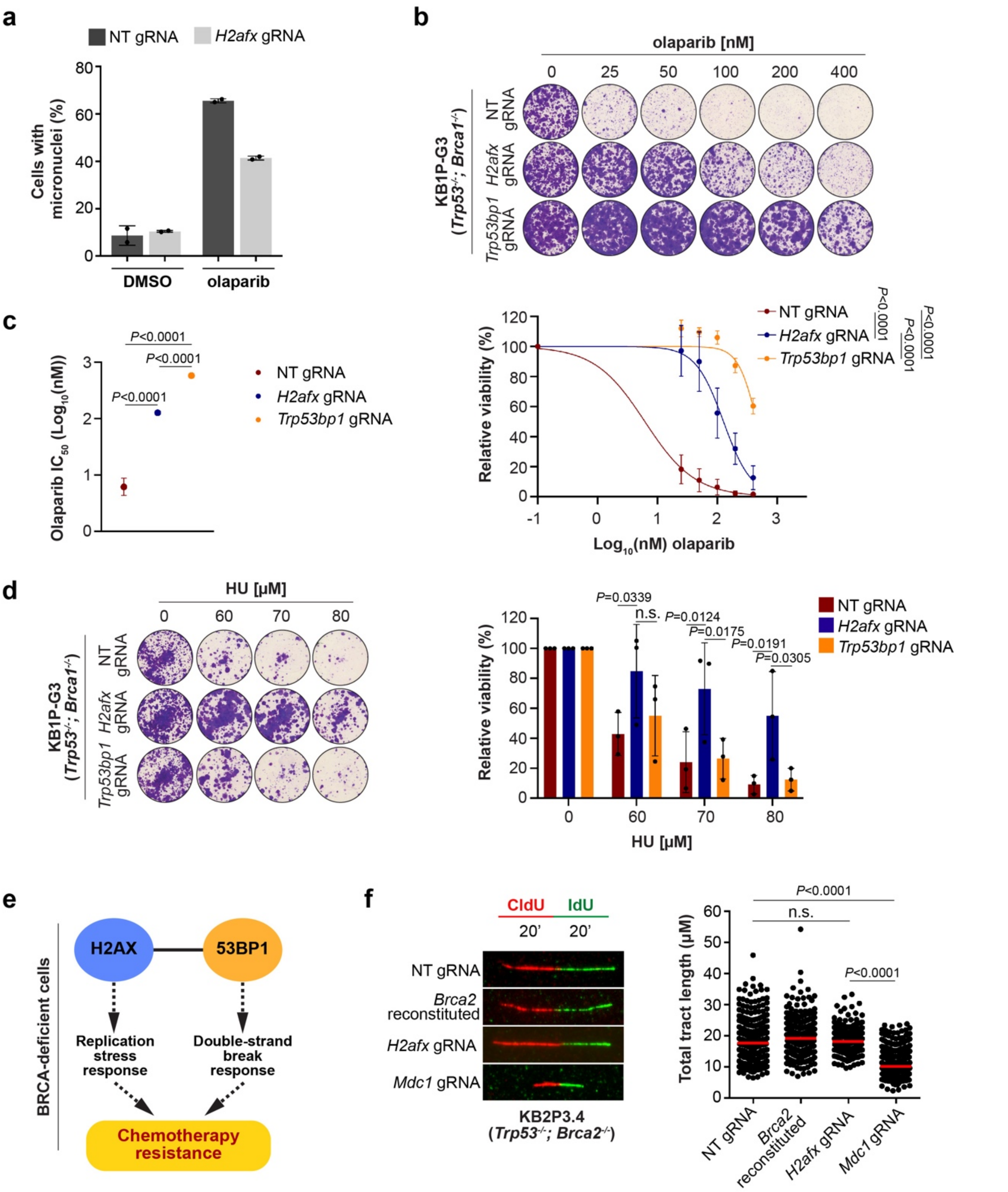

Supplementary Fig. 3. H2AX and 53BP1 control PARPi resistance through separable mechanisms. a, KB1P-G3-derived cells were treated with olaparib (3μM) for 24h. Plotted values are the mean±SD of micronucleated cells (n=2 independent experiments). **b**, Clonogenic survival assay of KB1P-G3-derived cells treated, or mock treated, with the indicated concentrations of olaparib for 12 days. Plotted values are the mean±SD clonogenic survival (n=3 independent experiments). *P* values were calculated with two-way Anova test and adjusted for multiple comparisons. **b**, Olaparib IC_{50} values were calculated for the indicated cell lines from the experiment performed in **b**. *P* values were calculated with two-way Anova

test and adjusted for multiple comparisons. **d**, Clonogenic survival assay of KB1P-G3-derived cells treated, or mock treated, with the indicated concentrations of hydroxyurea (HU) for 12 days. Plotted values are the mean±SD clonogenic survival (n=3 independent experiments). *P* values were calculated with two-way Anova test and adjusted for multiple comparisons. **e**, Schematic of the different mechanisms of resistance controlled by H2AX and 53BP1 in response to chemotherapy. **f,** DNA fiber analysis in KB2P3.4-derived cells treated according to the depicted scheme. Plotted values show the median of individual IdU/CldU ratios from at least 170 fibers (n=3 independent experiments). *P* values were calculated with one-way Anova test and adjusted for multiple comparisons.

Supplementary Fig. 4

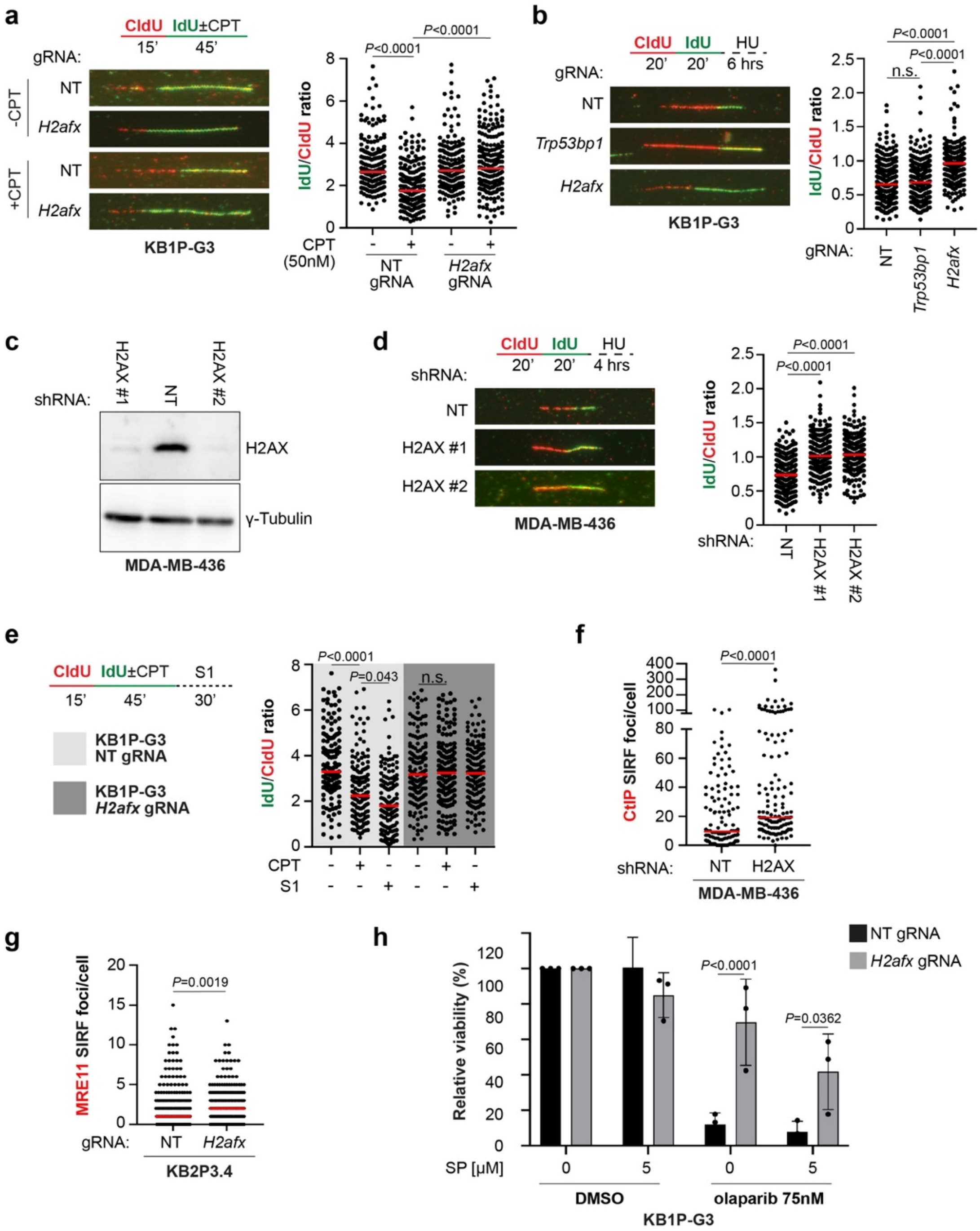

Supplementary Fig. 4. H2AX controls replication fork metabolism in BRCA-deficient mouse and human tumours. a, DNA fiber analysis in KB1P-G3-derived cells treated according to the depicted scheme. Plotted values show the median of individual IdU/CldU ratios from at least 180 fibers (n=3 independent experiments). *P* values were calculated with one-way Anova test and adjusted for multiple comparisons. **b,** DNA fiber analysis in KB1P-G3-derived cells treated according to the depicted scheme. Plotted values show the median of individual IdU/CldU ratios from 300 fibers (n=3 independent experiments). *P* values were calculated with one-way Anova test and adjusted for multiple comparisons. **c,** H2AX

immunoblotting in MDA-MB-436 cells expressing the indicated shRNA. Source data are provided as a Source Data file. **d,** DNA fiber analysis in MDA-MB-436-derived cells treated according to the depicted scheme. Plotted values show the median of individual IdU/CldU ratios from 300 fibers (n=3 independent experiments). *P* values were calculated with one-way Anova test and adjusted for multiple comparisons. **e,** DNA fiber analysis in KB1P-G3-derived cells treated according to the depicted scheme. Plotted values show the median of individual IdU/CldU ratios from at least 150 fibers (n=2 independent experiments). *P* values were calculated with one-way Anova test and adjusted for multiple comparisons. **f,** Plotted values show the median of CtIP SIRF foci from at least 150 cells (n=2 independent experiments). *P* values were calculated with one-way Anova test. **g,** Plotted values show the median of MRE11 SIRF foci from at least 300 cells (n=3 independent experiments). *P* values were calculated with one-way Anova test. **h,** Clonogenic survival assay of KB1P-G3-derived cells treated, or mock treated, with the indicated concentrations of SP and olaparib (75nM) for 12 days. Plotted values are the mean±SD clonogenic survival (n=4 independent experiments). *P* values were calculated with two-way Anova test and adjusted for multiple comparisons.

Supplementary Fig. 5

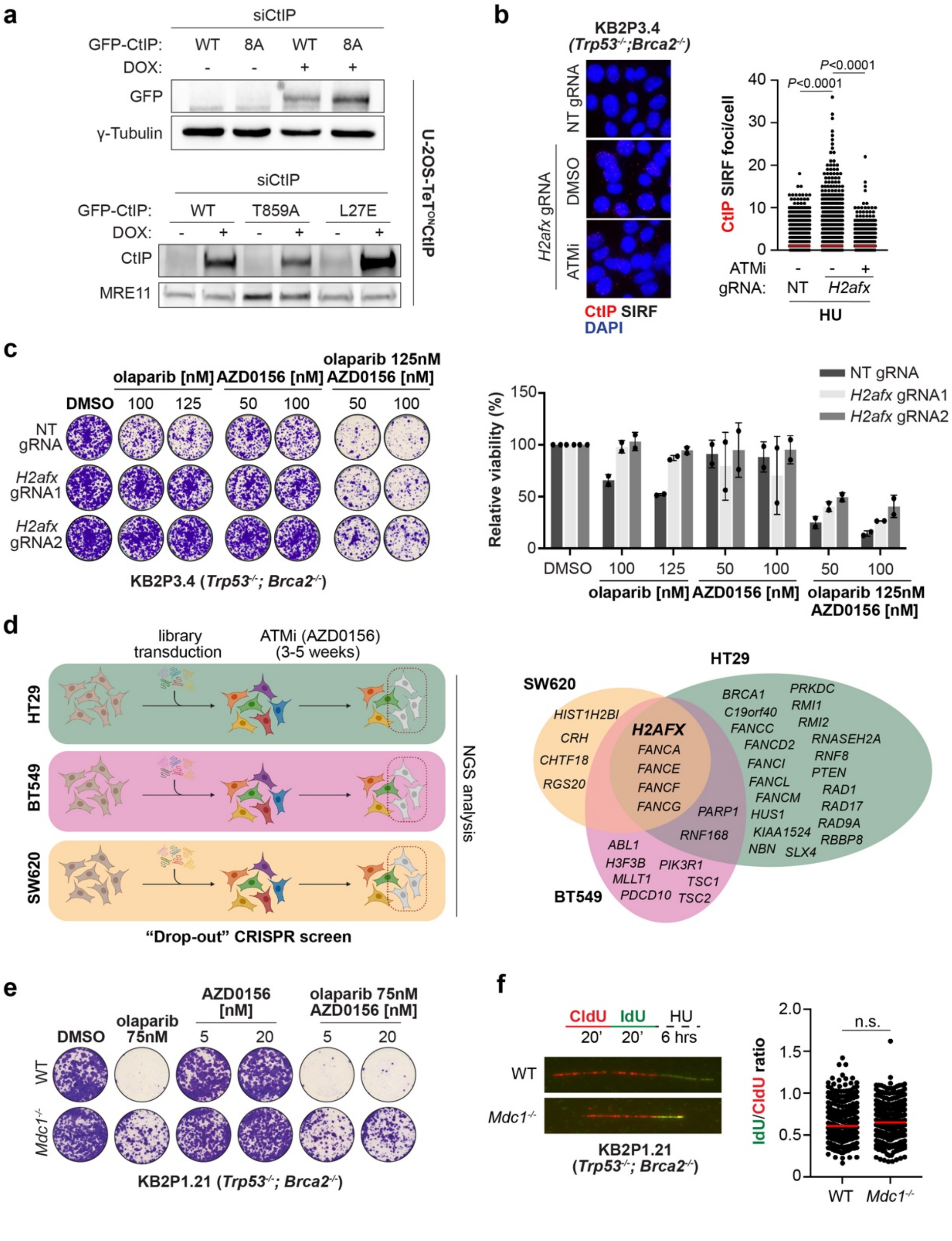

Supplementary data Fig. 5. Vulnerability to ATM inhibitors is an acquired trait of H2AX-deficient tumours. a, Immunoblot showing the expression of the different inducible GFP-CtIP constructs in U-2OS-TeT^{ON}-CtIP cells. Source data are provided as a Source Data file. **b,** Plotted values show the median of CtIP SIRF foci from at least 500 cells (n=3 independent experiments). *P* values were calculated with two-way Anova test and adjusted with the Tukey's test for multiple comparisons. **c,** Clonogenic survival assay of KB2P3.4-derived cells treated or mock treated with the indicated concentrations of AZD0156 and olaparib for 12 days. Plotted values are the mean±SD clonogenic survival (n=2 independent experiments). **d,** Design of the

functional CRISPR-Cas9 drop-out screen with the ATMi AZD0156 in distinct cancer cell lines. The Venn diagram shows the top hits from each individual CRISPR-Cas9 screen. **e,** Clonogenic survival assay of KB2P1.21 WT and *Mdc1*$^{-/-}$ cells treated or mock treated with the indicated concentrations of AZD0156 and olaparib (75nM) for 12 days. **f,** DNA fiber analysis in KB2P1.21 WT and *Mdc1*$^{-/-}$ cells treated according to the depicted scheme. Plotted values show the median of individual IdU/CldU ratios from 300 fibers (n=3 independent experiments). *P* values were calculated with one-way Anova test.

Uncropped blots related to Supplementary Fig. 1

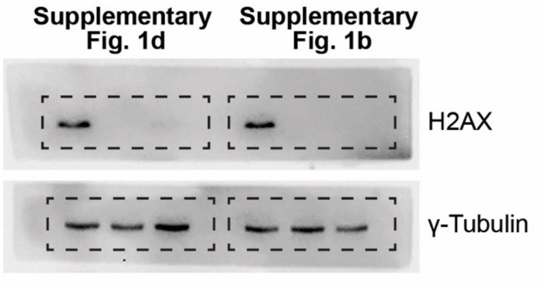

Uncropped blots related to Supplementary Fig. 2

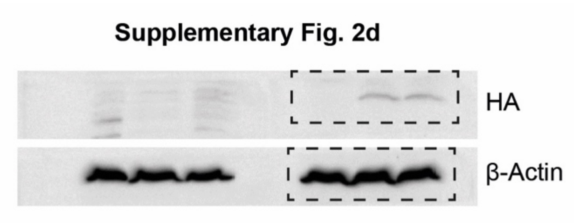

Uncropped blots related to Supplementary Fig. 4

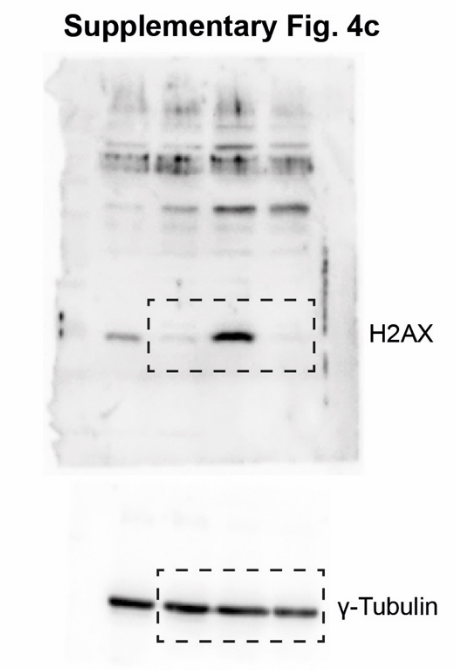

Uncropped blots related to Supplementary Fig. 5

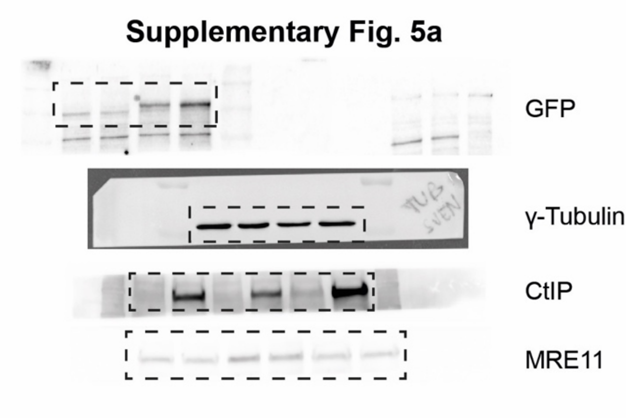

Supplement: Supplementary file 1 — Supplementary Information [file 41467_2024_48715_MOESM1_ESM.pdf]
