# Supplementary material for: H2AX promotes replication fork degradation and chemosensitivity in BRCA-deficient tumours
Source: Nat Commun. 2024 May 24;15:4430. doi: 10.1038/s41467-024-48715-1 (PMC11126719; doi:10.1038/s41467-024-48715-1)
Supplement: Supplementary file 2 — Reporting Summary [file 41467_2024_48715_MOESM2_ESM.pdf]

Corresponding author(s): Diego Dibitetto, Jos Jonkers, and Sven Rottenberg

Last updated by author(s): 05/07/2024

Reporting Summary

Nature Portfolio wishes to improve the reproducibility of the work that we publish. This form provides structure for consistency and transparency in reporting. For further information on Nature Portfolio policies, see our Editorial Policies and the Editorial Policy Checklist.

Statistics

For all statistical analyses, confirm that the following items are present in the figure legend, table legend, main text, or Methods section.

n/a	Confirmed	
☐	☒	The exact sample size (*n*) for each experimental group/condition, given as a discrete number and unit of measurement
☐	☒	A statement on whether measurements were taken from distinct samples or whether the same sample was measured repeatedly
☐	☒	The statistical test(s) used AND whether they are one- or two-sided<br>*Only common tests should be described solely by name; describe more complex techniques in the Methods section.*
☒	☐	A description of all covariates tested
☐	☒	A description of any assumptions or corrections, such as tests of normality and adjustment for multiple comparisons
☐	☒	A full description of the statistical parameters including central tendency (e.g. means) or other basic estimates (e.g. regression coefficient) AND variation (e.g. standard deviation) or associated estimates of uncertainty (e.g. confidence intervals)
☐	☒	For null hypothesis testing, the test statistic (e.g. *F*, *t*, *r*) with confidence intervals, effect sizes, degrees of freedom and *P* value noted<br>*Give P values as exact values whenever suitable.*
☒	☐	For Bayesian analysis, information on the choice of priors and Markov chain Monte Carlo settings
☒	☐	For hierarchical and complex designs, identification of the appropriate level for tests and full reporting of outcomes
☒	☐	Estimates of effect sizes (e.g. Cohen's *d*, Pearson's *r*), indicating how they were calculated

Our web collection on statistics for biologists contains articles on many of the points above.

Software and code

Policy information about availability of computer code

Data collection	SoftWoRx DeltaVision software, OpenLab, MAPS Version 3.14. For the shRNA and CRISPR screens, FASTA files were subjected to quality control with FASTQC and counted with the MaGeCK count function. Count tables were further supplied to algorithms described in the following section. Data from the RPE BRCA1 KO screen was downloaded from the online version of Noordermer et al. 2018 (https://static-content.springer.com/esm/art%3A10.1038%2Fs41586-018-0340-7/MediaObjects/41586_2018_340_MOESM4_ESM.xlsx
Data analysis	GraphPad Prism v7, ImageJ, Image Lab, MAPS Offline Viewer Version 3.14.11, softWoRx DeltaVision software, TIDE browser. Screen count tables were analyzed on UBELIX (http://www.id.unibe.ch/hpc), the HPC cluster at the University of Bern, using publicly available MaGeCK MLE module (Li et al. 2015). Results were processed in R open software (version 4.1.3) using the MaGeCKFlute package (Wang et al. 2019).

For manuscripts utilizing custom algorithms or software that are central to the research but not yet described in published literature, software must be made available to editors and reviewers. We strongly encourage code deposition in a community repository (e.g. GitHub). See the Nature Portfolio guidelines for submitting code & software for further information.

Data

Policy information about availability of data

All manuscripts must include a data availability statement. This statement should provide the following information, where applicable:

- Accession codes, unique identifiers, or web links for publicly available datasets
- A description of any restrictions on data availability
- For clinical datasets or third party data, please ensure that the statement adheres to our policy

All data will be shared upon request by the lead contact with no restrictions. Source data are provided with this paper. Raw sequencing data of the genetic screens are available in European Nucleotide Archive (ENA) under accession number PRJEB75036 (https://www.ebi.ac.uk/ena/browser/view/PRJEB75036) and PRJEB74933 (https://www.ebi.ac.uk/ena/browser/view/PRJEB74933). Raw sequencing data of WE-seq and RNA-seq reported in this paper are available in European Nucleotide Archive (ENA) under accession number PRJEB61242 (https://www.ebi.ac.uk/ena/browser/view/PRJEB61242) for KB1P(M) tumours and PRJEB61243 (https://www.ebi.ac.uk/ena/browser/view/PRJEB61243) for KB2P tumours. The remaining data are available within the Article, Supplementary Information, or Source Data file.

Research involving human participants, their data, or biological material

Policy information about studies with human participants or human data. See also policy information about sex, gender (identity/presentation), and sexual orientation and race, ethnicity and racism.

Reporting on sex and gender	There is no report on gender or sex.
Reporting on race, ethnicity, or other socially relevant groupings	There is no report on race, ethnicity or other socially elevant group.
Population characteristics	Clinical (diagnosis and past treatments), genomic informations and formalin-fixed paraffin embedded tumor sample were collected from an ovarian cancer patient with BRCA2 somatic mutation, from whom biopsies were available at diagnosis and when the patient aquired resistance to PARPi and chemotherapy. The age of the CHIOVAR59 patient was not disclosed to the authors of this work and it was not relevant for the purpose of the study.
Recruitment	Patients diagnosed with ovarian cancer at Hôpitaux Universitaires de Geneve were enrolled in the CHIOVAR biobank study.
Ethics oversight	The research protocol CHIOVAR was approved by the Commission Cantonale d'Ethique de la Recherche sur l'être humain (CCER 2018-00407).

Note that full information on the approval of the study protocol must also be provided in the manuscript.

Field-specific reporting

Please select the one below that is the best fit for your research. If you are not sure, read the appropriate sections before making your selection.

[x] Life sciences [ ] Behavioural & social sciences [ ] Ecological, evolutionary & environmental sciences

For a reference copy of the document with all sections, see nature.com/documents/nr-reporting-summary-flat.pdf

Life sciences study design

All studies must disclose on these points even when the disclosure is negative.

Sample size	All experiments represented in this manuscript are performed as biological replicates, with at least two or three independent replicates, as mentioned in each figure legend. For the in vivo transplantation studies, the number of mice for each experiment was calculated based on power analysis performed in previous publication from the lab. The number of biological replicates and the number of individual measurements (DNA fibers or foci) were considered to have enough power to reveal relevant biological insights with sufficient statistical significance.
Data exclusions	No data were excluded.
Replication	For each experiment we performed independent experiments to ensure reproducibility of the observed results. Description of the exact number of independent repetitions (n) is indicated in each corresponding figure legend. All attempts were succesful. In some cases,replicates were performed by different operators to increase confidence on the obtained results.
Randomization	For the in vivo transplantation experiments, mice were stratified into the different treatment arms by random allocation into the vehicle, olaparib or ATMi treated group. Randomization was not necessary to any other experiment due to the relatively small sample size.
Blinding	For the in vivo experiments, treatment of mice with the modified tumours was performed blind. Blinding was not applied to any other experiment. Unbiased results were obtained from the combination of multiple repetitions from independent researchers.

Reporting for specific materials, systems and methods

We require information from authors about some types of materials, experimental systems and methods used in many studies. Here, indicate whether each material, system or method listed is relevant to your study. If you are not sure if a list item applies to your research, read the appropriate section before selecting a response.

Materials & experimental systems

n/a	Involved in the study
☐	☒ Antibodies
☐	☒ Eukaryotic cell lines
☒	☐ Palaeontology and archaeology
☐	☒ Animals and other organisms
☒	☐ Clinical data
☒	☐ Dual use research of concern
☒	☐ Plants

Methods

n/a	Involved in the study
☒	☐ ChIP-seq
☒	☐ Flow cytometry
☒	☐ MRI-based neuroimaging

Antibodies

Antibodies used	Primary antibodies<br>mouse anti-HA (1:1,000, #901533, Biolegend)<br>mouse anti-γ-Tubulin (1:1,000, #5886, Cell Signalling)<br>rabbit anti-GFP (1:1,000, #2555, Cell Signalling)<br>rabbit anti-53BP1 (1:800, #A300-272A, Bethyl laboratories)<br>rabbit anti-RAD51 (1:1,000, #70-012, Bioacademia)<br>rat anti-BrdU (CldU) (1:250, #ab6326, Abcam)<br>mouse anti-BrdU (IdU) (1:40, #347580, BD Biosciences)<br>rabbit anti-CtIP (1:500, #A300-487A, Bethyl Laboratories)<br>mouse anti-Biotin (1:200, #200-002-211, Jackson Immuno Research)<br>rabbit anti-MRE11 (1:500, a kind gift from Arnab Ray Chaudhuri)<br>rabbit anti-H2AX (1:1000, #NB100-383, Novus Biologicals)<br><br>Secondary antibodies<br>anti-rabbit Horseradish Peroxidase (HRP)-linked secondary antibodies (1: 5,000, Cell Signalling)<br>anti-mouse Horseradish Peroxidase (HRP)-linked secondary antibodies (1: 5,000, Cell Signalling)<br>Alexa Fluor 488 goat anti-mouse (#A-11029, ThermoFisher Scientific, 1:600)<br>Cy3 AffiniPure $F(ab')_2$ fragment donkey anti-rat (#712-165-513, Jackson Immuno Research, 1:150)<br>Alexa Fluor 568 goat anti-rabbit (1:2,000, # T-6391, ThermoFisher Scientific)
Validation	All antibodies used are commercially available and have been previously validated by the corresponding manufacturers as stated in their websites. Antibodies used for SIRF experiments have been tested in our lab individually in "single antibody" and "no EdU" control samples. Anti-H2AX antibody for IHC has been further validated by the Translational Research Unit at the University of Bern.

Eukaryotic cell lines

Policy information about cell lines and Sex and Gender in Research

Cell line source(s)	The original mouse cell lines used in this study have been previously described (Evers et al. 2008; Jaspers et al. 2013). RPE TP53KO; BRCA1KO were a gift from the Durocher lab. SW620, HT29, and BT549 were purchased from ATCC.
Authentication	All mouse cell lines were authenticated by Brca1/2-specific PCR-based genotyping (mouse) (Evers et al. 2008; Jaspers et al. 2013).
Mycoplasma contamination	Cells have been tested negative for mycoplasma contamination.
Commonly misidentified lines (See ICLAC register)	None of the used cell lines are listed in the ICLAC database.

Animals and other research organisms

Policy information about studies involving animals; ARRIVE guidelines recommended for reporting animal research, and Sex and Gender in Research

Laboratory animals	NMRI-nude female mice were purchased from Janvier Laboratories and used for in vivo studies at the age of 6-9 weeks.<br>KB1P and KB2P mice strains were previously described (Evers et al. 2008; Bouwman et al. 2013; Duarte et al. 2017; Gogola et al.

	2018). All mice were housed on standard 12 hour day/night cycle in ventilated cages (4 animals/cage) with ad libitum food. Room temperature was maintained at 21 degrees Celsius and humidity was 55%. The age of the experimental animals is indicated in the methods section.
Wild animals	No wild animals were used in this study.
Reporting on sex	All experiments were performed with female mice as this work focuses on breast cancer.
Field-collected samples	The study did not involve samples collected from the field.
Ethics oversight	All animal experiments were approved by the Animal Ethics Committee of The Netherlands Cancer Institute (Amsterdam, the Netherlands) and the Canton of Bern (license BE69/2021) and were performed in full compliance with national laws, which enforce Dir. 2010/63/EU (Directive 2010/63/EU of the European Parliament and of the Council of 22 September 2010 on the protection of animals used for scientific purposes).

Note that full information on the approval of the study protocol must also be provided in the manuscript.
